# Probability of entering an orthant by correlated fractional Brownian motion with drift: exact asymptotics

**DOI:** 10.1007/s10687-024-00489-x

**Published:** 2024-08-29

**Authors:** Krzysztof Dȩbicki, Lanpeng Ji, Svyatoslav Novikov

**Affiliations:** 1 Mathematical Institute, University of Wrocław, pl. Grunwaldzki 2/4, 50-384, Wrocław, Poland; 2 School of Mathematics, University of Leeds, Woodhouse Lane, LS2 9JT Leeds, United Kingdom; 3 Department of Actuarial Science, University of Lausanne, UNIL-Dorigny, 1015 Lausanne, Switzerland

**Keywords:** Multi-dimensional fractional Brownian motion, Extremes, Exact asymptotics, Large deviations, Quadratic programming problem, Dimension reduction, Primary—60G15, Secondary—60G70

## Abstract

For $\{\varvec{B}_{H}(t)= (B_{H,1}(t) ,\ldots ,B_{H,d}(t))^{{\top }},t\ge 0\}$, where $\{B_{H,i}(t),t\ge 0\}, 1\le i\le d$ are mutually independent fractional Brownian motions, we obtain the exact asymptotics of

$$\mathbb P (\exists t\ge 0: A \varvec{B}_{H}(t) - \varvec{\mu }t >\varvec{\nu }u), \ \ \ \ u\rightarrow \infty ,$$

where *A* is a non-singular $d\times d$ matrix and $\varvec{\mu }=(\mu _1,\ldots , \mu _d)^{{\top }}\in \mathbb {R}^d$, $\varvec{\nu }=(\nu _1, \ldots , \nu _d)^{{\top }} \in \mathbb {R}^d$ are such that there exists some $1\le i\le d$ such that $\mu _i>0, \nu _i>0.$

## Introduction

Consider a vector-valued Gaussian process $\{{\varvec{X}}(t),t\ge 0\}$, where ${\varvec{X}}(t)=A \varvec{B}_{H}(t)$ with $A\in \mathbb {R}^{d\times d}$ a non-singular matrix and $\{\varvec{B}_{H}(t)= (B_{H,1}(t) ,\ldots ,B_{H,d}(t))^{{\top }},t\ge 0\}$ with $\{B_{H,i}(t),t\ge 0\}, 1\le i\le d$ ($d\in \mathbb {N}$) being mutually independent fractional Brownian motions (fBms), i.e., centered Gaussian processes with stationary increments, continuous sample paths and variance functions $\text {Var}(B_{H,i}(t))=t^{2H}$, $H\in (0,1)$.

We focus on the exact asymptotic behavior of the probability that a drifted correlated fractional Brownian motion $\varvec{X}$ enters orthant $\mathcal {O}_u=\{(x_1 ,\ldots ,x_d)^{{\top }}: x_i> \nu _i u, i=1 ,\ldots ,d\}$ over an infinite-time horizon, i.e.,

1 $$\begin{aligned} P(u):=\mathbb {P} \left( \exists t\ge 0: \varvec{X}(t) - \varvec{\mu }t \ {\in \mathcal {O}_u} \right) =\mathbb {P} \left( \exists _{t \ge 0} \forall _{i=1 ,\ldots ,d} X_{i}(t) -\mu _i t> \nu _iu \right) , \end{aligned}$$

as $u\rightarrow \infty$ for $\varvec{\mu }=(\mu _1,\ldots , \mu _d)^{{\top }}\in \mathbb {R}^d$ and $\varvec{\nu }=(\nu _1, \ldots , \nu _d) ^{{\top }}\in \mathbb {R}^d$.

We are interested in the case that the above probability is a rare event, that is, $P(u)\rightarrow 0$ as $u\rightarrow \infty$, for which we shall assume that there exists some $1\le i\le d$ such that $\mu _i>0, \nu _i>0.$

The probability *P*(*u*) defined in Eq. (1) is of interest both for theory-oriented studies and for applied-mathematics problems. One of important motivations to analyze Eq. (1) stems from *ruin theory*, where *P*(*u*) describes simultaneous ruin probability in infinite-time horizon of *d* dependent business lines whose risk processes $R_i(t),t\ge 0$ are modeled by

$$R_i(t)=\nu _iu +\mu _i t -X_{i}(t),$$

where $\nu _iu$ represents the initial capital, $\mu _i$ is the net profit rate and $X_{i}(t)$ is the net loss up to time *t*; we refer to Michna (1998) for the formal justification of the use of fractional Brownian motion to model the risk process.

In the 1-dimensional case, $d=1$, the exact asymptotics for *P*(*u*) was derived in the seminal paper by Hüsler and Piterbarg (1999); see also Dȩbicki (2002); Hüsler and Piterbarg (2004); Dieker (2005) for extensions to other classes of stochastic processes with stationary increments.

In the multidimensional case, the exact asymptotics of *P*(*u*) as $u\rightarrow \infty$ is known only for the special Brownian model, i.e., when $H=1/2$; see Dȩbicki et al. (2018). The strategy of the proof there, although in its roots based on *the double sum* technique developed in the 1-dimensional setting for extremes of Gaussian processes (see, e.g. Pickands 1969, 1967; Piterbarg 1996), needed new ideas that in several key steps of the argumentation significantly differ from methods used in the 1-dimensional case. In particular, one of the difficulties is the lack of Slepian-type inequalities that could be applied in the multidimensional setting, which was overcome in Dȩbicki et al. (2018) by the heavy use of the independence of increments property of Brownian motion.

In this contribution, we aim to complement the findings of Dȩbicki et al. (2018) by tackling the fBm problem (1) for $H\ne 1/2$. Interestingly, in contrary to the Brownian case, the full analysis of all the cases needs to consider two separate scenarios described by the local behaviour of the function

$$\varvec{\zeta }(t)=(\zeta _1(t), \ldots , \zeta _d(t))^{{\top }} := (\varvec{\nu }+\varvec{\mu }t)/t^H, t>0$$

in the neighbourhood of the unique point $t_0$ that minimizes function

2 $$\begin{aligned} g(t):=\frac{1}{t^{2H}} \inf _{\varvec{v} \ge \varvec{\nu }+\varvec{\mu } t} \varvec{v}^\top (AA^\top )^{-1} \varvec{v} \end{aligned}$$

over $t\ge 0$, where the point $t_0u$ has a natural interpretation as the *most probable time* for the process $\{\varvec{X}(t) - \varvec{\mu }t,t\ge 0\}$ to enter orthant $\mathcal {O}_u$; see also Section 2. Let $I\subset \{1,...,d\}$ be the set of coordinates that contribute to the asymptotics of *P*(*u*), as $u\rightarrow \infty$; see Section 2 for the details about how to specify *I*.

In the first scenario, when $H<{1}/{2}$, or $H>1/2$ and $\zeta ^{'}_i(t_0)={0}$ for all $i\in I$, the local steepness of the correlation function of $X_i$ is higher than the local steepness of $\zeta _i(t)$ in the neighbourhood of the point $t_0$. This case can be solved by an adaptation of the technique developed in Dȩbicki et al. (2020) for extremes of centered vector-valued Gaussian processes over a finite time horizon.

The complementary case, $H>1/2$ but $\zeta ^{'}_i(t_0)\ne {0}$ for some $i\in I$, is different from the previous one since for those coordinates $i\in I$ for which $\zeta ^{'}_i(t_0)\ne {0}$ the local steepness of the correlation function of $X_i$ is lower than the local steepness of $\zeta _i(t)$ in the neighbourhood of the point $t_0$. This scenario needs a novel approach which leads also to a different asymptotics of *P*(*u*) as $u\rightarrow \infty$.

The results derived in this contribution go in line with recent findings on the tail asymptotics of extremes of vector-valued Gaussian processes, where most of the available literature deals with centered marginal processes or over a compact parameter space (Dȩbicki et al. 2020; Bisewski et al. 2023; Ievlev 2024; Ievlev and Kriukov 2024; Cheng and Xiao 2023).

### Notation

We shall use some standard notation which is common when dealing with (column) vectors. All the operations on vectors are meant componentwise, for instance, for any given $\varvec{x} = (x_1,\ldots ,x_d)^{{\top }}\in \mathbb {R}^d$ and $\varvec{y} = (y_1,\ldots ,y_d)^{{\top }} \in \mathbb {R}^d$, we write $\varvec{x} > \varvec{y}$ if and only if $x_i > y_i$ for all $1 \le i \le d$, write $1/\varvec{x}=(1/x_1,\cdots ,1/x_d)^{{\top }}$ if $x_i\ne 0, 1 \le i \le d$, and write $\varvec{x}\varvec{y}=(x_1y_1 ,\ldots ,x_dy_d)^{{\top }}$ and $a\varvec{x} =(a x_1,\ldots , ax_d)^{{\top }},$ $a\in \mathbb {R}.$ Further, we set $\varvec{0}:= (0,\ldots ,0)^{{\top }}$ and $\varvec{1}: = (1,\ldots ,1)^{{\top }}$ whose dimension will be clear from the context. Moreover, denote $\left|\varvec{x} \right|$ as the $L_1$-norm of $\varvec{x} = (x_1,\ldots ,x_d)^{{\top }}\in \mathbb {R}^d$.

If $I\subset \{1,\ldots ,d\}$, then for a vector $\varvec{a}\in \mathbb {R}^d$ we denote by $\varvec{a}_I=(a_i, i\in I)$ a sub-block vector of $\varvec{a}$. Similarly, if further $J \subset \{1,\ldots ,d\}$, for a matrix $M=(m_{ij})_{i,j\in \{1,\ldots ,d\}}\in \mathbb {R}^{d\times d}$ we denote by $M_{IJ}=(m_{ij})_{i\in I, j\in J}$ the sub-block matrix of *M* determined by *I* and *J*. Further, write $M_{II}^{-1}=(M_{II})^{-1}$ for the inverse matrix of $M_{II}$ whenever it exists. Denote by $\left|I \right|$ the number of elements in the index set *I* and by $\left|M \right|$ the determinant of a square matrix *M*.

For two positive functions *f*, *h* and some $u_0\in [-\infty , \infty ]$, write $f(u) = h(u)(1+o(1))$ or $h(u)\sim f(u)$ if $\lim _{u \rightarrow u_0} f(u) /h(u) = 1$, write $f(u) = o(h(u))$ if $\lim _{u \rightarrow u_0} {f(u)}/{h(u)} = 0$. Moreover, for an event *E* we denote $\mathbb {I}_{(E)}$ as the indicator function of *E*.

### Organization of the paper

Some preliminary results related with properties and the role of function *g* defined in Eq. (2) are presented in Section 2. The main result of this contribution, which is Theorem 3.1, is given in Section 3, followed by an illustrative example. In Section 4, we give the proof of the main result. All other technical proofs are relegated to Appendix.

##  Preliminary results

It is known that the approximation of the probability *P*(*u*) depends on the solution of a related quadratic optimization problem. In particular, in the light of (Dȩbicki et al. (2010), Theorem 1), the logarithmic asymptotics can be derived and takes the following form

3 $$\begin{aligned} -\ln P(u) \sim \frac{\widehat{g}}{2} u^{2(1-H)}, \quad \text {with}\ \ \widehat{g}=\inf _{t\ge 0} g(t), \end{aligned}$$

where

4 $$\begin{aligned} g(t)=\frac{1}{t^{2H}} \inf _{\varvec{v} \ge \varvec{\nu }+\varvec{\mu } t} \varvec{v}^\top \Sigma ^{-1} \varvec{v}, \quad \Sigma =AA^\top . \end{aligned}$$

The properties of function *g*, in particular, the existence of its minimizer and expansions in the neighbourhood of this point (when exists) are crucial to the exact asymptotic analysis. In order to introduce some further notation and for further reference, we present a lemma on the quadratic optimization problem stated in Hashorva (2005) (see also Hashorva and Hüsler 2002).

### Lemma 2.1

Let $\Sigma \in \mathbb {R}^{d \times d},d\ge 2$ be a positive definite matrix. If $\varvec{b}\in \mathbb {R}^d \setminus (-\infty , 0]^d$, then the quadratic programming problem

$$P_\Sigma (\varvec{b}): \text {minimize } \varvec{x}^\top \Sigma ^{-1} \varvec{x} \textrm{under the linear constraint } \varvec{x} \ge \varvec{b}$$

has a unique solution $\widetilde{\varvec{b}}$ and there exists a unique non-empty index set $I\subseteq \{1, \ldots , d\}$ such that

5 $$\begin{aligned} & \widetilde{\varvec{b}}_{I}= \varvec{b}_{I}\not = \varvec{0}_I, \quad \widetilde{\varvec{b}}_{I^c} = \Sigma _{I^cI}\Sigma _{II}^{-1} \varvec{b}_{I}\ge \varvec{b}_{I^c}, \quad \Sigma _{II}^{-1} \varvec{b}_{I}>\varvec{0}_I,\end{aligned}$$

6 $$\begin{aligned} & \min _{\varvec{x} \ge \varvec{b}} \varvec{x}^\top \Sigma ^{-1}\varvec{x}= \widetilde{\varvec{b}}^\top \Sigma ^{-1} \widetilde{\varvec{b}} = \varvec{b}_{I}^\top \Sigma _{II}^{-1}\varvec{b}_{I}>0, \end{aligned}$$

where $I^c= \{1, \ldots , d\} \setminus I.$ Moreover, denoting $\varvec{w}=\Sigma ^{-1} \widetilde{\varvec{b}}$ we have

7 $$\begin{aligned} \varvec{w}_I=\Sigma _{II}^{-1} \varvec{b}_{I}>\varvec{0}_I, \quad \varvec{w}_{I^c} =\varvec{0}_{I^c}. \end{aligned}$$

The next lemma includes some properties of the function *g* and its relative $g_I(t)=\frac{1}{t^{2H}} (\varvec{\nu }+\varvec{\mu }t)^\top _{I} \Sigma _{II}^{-1} (\varvec{\nu }+\varvec{\mu }t)_I$ with *I* the index set as in Lemma 2.1. We defer its proof to Appendix.

### Lemma 2.2

Function $g\in C^1(0,\infty )$ and achieves its unique minimum at

8 $$\begin{aligned} t_{0}=\frac{\sqrt{4 (\varvec{\nu }_{I}^\top \Sigma ^{-1}_{II} \varvec{\mu }_{I} )^2(1-2H)^2 +16 H(1-H) \varvec{\nu }_{I}^\top \Sigma ^{-1}_{II} \varvec{\nu }_{I} \varvec{\mu }_{I}^\top \Sigma ^{-1}_{II} \varvec{\mu }_{I} }- 2 (1-2H) \varvec{\nu }_{I}^\top \Sigma ^{-1}_{II} \varvec{\mu }_{I}}{4 \varvec{\mu }_{I}^\top \Sigma ^{-1}_{II} \varvec{\mu }_{I} (1-H)} >0 \end{aligned}$$

with

9 $$\begin{aligned} g(t_0)=\inf _{t>0}\frac{1}{t^{2H}} \inf _{\varvec{v} \ge \varvec{\nu }+\varvec{\mu } t} \varvec{v}^\top \Sigma ^{-1} \varvec{v}=\frac{1}{t_0^{2H}} \varvec{b}^\top _{I} \Sigma _{II}^{-1} \varvec{b}_{I}=g_I(t_0), \end{aligned}$$

where

$$\varvec{b}=\varvec{b}(t_0), \quad \text {with \ } \varvec{b}(t)= \varvec{\nu }+ \varvec{\mu }t,$$

and $I=I(t_0)$ being the index set corresponding to the solution of $P_\Sigma (\varvec{b})$. Moreover,

10 $$\begin{aligned} g_I(t_0+ t) = g_I(t_0)+ \frac{g''_I(t_0)}{2} t^2(1+o(1)), \ \ \ \ t\rightarrow 0, \end{aligned}$$

where

$$g''_I(t_0)=\frac{1}{t_0^{2H+1}}\left( 4 \varvec{\mu }_{I}^\top \Sigma ^{-1}_{II} \varvec{\mu }_{I} (1-H) t_0 +2(1-2H)\varvec{\nu }_{I}^\top \Sigma ^{-1}_{II} \varvec{\mu }_{I}\right) >0.$$

### Remark 2.3

Note that $t_0$ given in Eq. (8) is actually an equation of $t_0$ because $I=I(t_0)$ is a set function of $t_0$. Here, for any fixed $t>0,$ $I(t)\subseteq \{1,2,\ldots , d\}$ is the index set of the solution to the quadratic programming problem $P_{\Sigma }(\varvec{b}(t))$, see Lemma 2.1. We remark that, for specific problems, both the index set *I* and $t_0$ can be identified explicitly; see Example 3.3 in Section 3 or the examples presented in Dȩbicki et al. (2018).

Hereafter we shall use the notation $\varvec{b}=\varvec{b}(t_0)= \varvec{\nu }+ \varvec{\mu }t_0$, and $I=I(t_0)$ for the *essential* index set of the quadratic programming problem $P_{\Sigma }(\varvec{b})$. Furthermore, let $\widetilde{\varvec{b}}$ be the unique solution of $P_{\Sigma }(\varvec{b})$. If $I^c=\{1,\ldots ,d\}\setminus I\ne \emptyset$, we define the *weakly essential index* and the *unessential index* sets by

11 $$\begin{aligned} K=\{j\in I^c: \widetilde{b}_j=\Sigma _{jI}\Sigma _{II}^{-1}\varvec{b}_I=b_j\}, \quad \text { and } J=\{j\in I^c: \widetilde{b}_j=\Sigma _{jI}\Sigma _{II}^{-1}\varvec{b}_I>b_j\}, \end{aligned}$$

respectively.

## Main result

In this section we present the main result of this contribution. Recall that through the whole paper we assume that there exists some $1\le i\le d$ such that $\mu _i>0, \nu _i>0.$ Denote $\varvec{W}_{I}(t) =D \varvec{B}_{H,I}(t)$, with *D* a matrix such that $D D^\top = \Sigma _{II}$. We define a *generalized Pickands constant* as

$$\begin{aligned} \mathcal {H}_{I}= \lim _{T\rightarrow \infty } \frac{1}{T} \mathcal {H}_{I}(T) \in (0,\infty ), \end{aligned}$$

where

$$\begin{aligned} \mathcal {H}_{I}(T)= \int _{\mathbb {R}^{\left|I \right|}} e^{\frac{1}{t_0^{2H}} \varvec{w}_I^\top \varvec{x}_I} \mathbb {P} \left( \exists _{t \in [0, T]} \varvec{W}_{I}(t) - \frac{1}{2t_0^{2H}}\varvec{b}_I t^{2H}>\varvec{x}_I \right) d\varvec{x}_I, \end{aligned}$$

with $\varvec{b} = \varvec{\nu }+ \varvec{\mu }t_0$ and $\varvec{w}_I =\Sigma _{II}^{-1} \varvec{b}_I.$ We remark that $\mathcal {H}_I$ is well-defined, finite and positive, since it is a multiple of the multidimensional Pickands constant $\mathcal {H}_{2H,V}$ defined in (2.5) of Dȩbicki et al. (2020) with $V=\left(2 t_0^{4H}\right)^{-1} \textrm{diag}(\varvec{w}_I) \Sigma _{II} \textrm{diag}(\varvec{w}_I)$.

For *K* defined in Eq. (11), let

12 $$\begin{aligned} & {\mathcal {C}_{K}}:= \left\{ \begin{array}{lll} {\frac{1}{\sqrt{ (2\pi t_0^{2H} )^{\left|I \right|} \left|\Sigma _{II} \right|}} } \int _{\mathbb {R}} e^{-\frac{1}{4} g_I''(t_0) y^2} \ \cdot \mathbb {P} \left( \varvec{Y}_K< t_0^{-H} \left(\varvec{\mu }_K - \Sigma _{KI}\Sigma _{II}^{-1} \varvec{\mu }_I\right) y \right) dy,\\ & K\ne \emptyset ,\\ {\sqrt{ \frac{4\pi }{g''_I(t_0) (2\pi t_0^{2H} )^{\left|I \right|} \left|\Sigma _{II} \right|} } }, & K=\emptyset . \end{array}\right. \end{aligned}$$

where $$\varvec{Y}_K \overset{d}{\sim }\mathcal {N}\left(\varvec{0}_K, \Sigma _{KK} - \Sigma _{KI}\Sigma _{II}^{-1} \Sigma _{IK} \right)$$.

Following Section 2, the logarithmic asymptotics of *P*(*u*) as $u\rightarrow \infty$ depends on $t_0$, the minimizer of function *g* defined in Eq. (4). As stated in the following theorem, the exact asymptotics of *P*(*u*) splits on two scenarios. In order to catch an intuition behind this division, for a while let us consider the 1-dimensional problem $\mathbb {P} \left( \exists _{t \ge 0} B_{H,i}(t) -\mu _i t>\nu _i u \right) ,$ assuming $\mu _i,\nu _i>0.$ By self-similarity of $B_{H,i}$ we get

$$\begin{aligned} \mathbb {P} \left( \exists _{t \ge 0} B_{H,i}(t) -\mu _i t>\nu _i u \right) = \mathbb {P} \left( \exists _{t \ge 0} \frac{B_{H,i}(t)}{\nu _i u +\mu _i t}>1 \right) = \mathbb {P} \left( \exists _{t \ge 0} \frac{B_{H,i}(t)}{\nu _i +\mu _i t}>u^{1-H} \right) \end{aligned}$$

and thus, following the same lines of reasoning as in Section 2 but for 1-dimensional setting, the logarithmic asymptotics of the above is determined by $t_{0,i}$, the unique minimizer of $\zeta _i(t)=\frac{\nu _i +\mu _i t}{t^H}$, $t>0$, that is the point that satisfies ${\zeta _i}'({t_{0,i}}) = {0}$ or equivalently

13 $$\begin{aligned} H {\nu _i} = (1-H) {t_{0,i}} {\mu _i}.\end{aligned}$$

This leads to two cases, where the play between the value of the optimizing point $t_0$ and the optimizers $t_{0,i}$ of $\zeta _i(t)$ for $i\in I$ is crucial:

$\diamond$ $H<{1}/{2}$, or $H>1/2$ and $\zeta ^{'}_i(t_0)={0}$ for all $i\in I$. Then the local steepness of the correlation function of $X_i$ is higher than the local steepness of $\zeta _i(t)$ in the neighbourhood of the point $t_0$.

$\diamond$ $H>1/2$ but $\zeta ^{'}_i(t_0)\ne {0}$ for some $i\in I$. For the coordinates $i\in I$ for which $\zeta ^{'}_i(t_0)\ne {0}$ the local steepness of the correlation function of $X_i$ is lower than the local steepness of $\zeta _i(t)$ in the neighbourhood of the point $t_0$.

The following theorem constitutes the main finding of this contribution. Let us recall notation $x_{-}=\max (0,-x),\,x \in \mathbb {R}$.

### Theorem 3.1

(i). If $H<{1}/{2}$, or $H>1/2$ and $H \varvec{\nu }_{I} = (1-H) t_0 \varvec{\mu }_{I}$, then
  $$\begin{aligned} P(u) \sim \mathcal {H}_{I}{\mathcal {C}_{K}} u^{-\left|I \right|(1-H)+1/H+H-2} e^{-\frac{g_I(t_0)}{2}u^{2(1-H)} },\ \ u\rightarrow \infty . \end{aligned}$$
(ii). If $H>1/2$ but $H\varvec{\nu }_{I} \ne (1-H) t_0\varvec{\mu }_{I}$, then
  $$\begin{aligned} P(u) \sim {\frac{t_0^{2H(\left|I \right|-1)}}{\prod _{i\in I}w_i} \mathcal {C}_{K}} \left(\sum _{i \in I} \left( w_i \mu _i - \frac{H}{t_0} w_i b_i\right) _{-} \right) u^{-\left|I \right|(1-H)+1-H} e^{-\frac{g_I(t_0)}{2}u^{2(1-H)} }, \ \ u\rightarrow \infty , \end{aligned}$$
  where
  $$\sum _{i \in I} \left( w_i \mu _i - \frac{H}{t_0} w_i b_i\right) _{-} >0.$$

### Remark 3.2

We remark on the role of the index set *J* played in the asymptotics when it is non-empty. It is concluded from Dȩbicki et al. (2018) that the index set *J* and the corresponding components do not play any role in the exact asymptotics of *P*(*u*) for $H=1/2$. It follows from Theorem 3.1 that the same observation applies also for $H \ne 1/2$.

We conclude this section with an illustrative example, where we will see how the index sets *I*, *K*, *J* and the optimal point $t_0$ are derived and how the different cases may appear. Our purpose of this example is not to be as general as possible, but to be restrictive so that it includes an interesting scenario.

### Example 3.3

We consider a 4-dimensional Gaussian process with independent (positive or negative) drifted fBm components. Precisely, let

$$d=4,\ \ \Sigma =\textrm{Id},\ \ \nu _i>0, i=1,2,3,4, \ \ \mu _1, \mu _2>0,\ \ \mu _3, \mu _4<0.$$

We also assume that

14 $$\begin{aligned} \infty =:t'_3>t'_2:=\frac{\nu _3}{\left|\mu _3 \right|}> \frac{\nu _4}{\left|\mu _4 \right|}=:t'_1 >t'_0:=0. \end{aligned}$$

Denote

$$I_1=\{1,2,3,4\}, \ \ I_2=\{1,2,3\}, \ \ I_3=\{1,2\}, \ \ I^c_j=\{1,2,3,4\}\setminus I_j,\ j=1,2,3.$$

It can be seen that

$$\varvec{\nu }_{I_j} +t \varvec{\mu }_{I_j} >\varvec{0}_{I_j},\ \ \varvec{\nu }_{I_j^c} +t \varvec{\mu }_{I_j^c} \le \varvec{0}_{I_j^c}, \ \ \ \ t\in [t'_{j-1}, t'_j)$$

and thus, by Lemma 2.1,

$$I(t) = I_j, \ t\in [t'_{j-1},t'_j), \ \ \ \ j=1,2,3.$$

Further, it follows from Lemma 2.2 that the optimal point $t_0$ is equal to the $t_0^{(k)}$ defined as Eq. (8) with $I_k$, such that (see Eq. (9))

$$g_{I_k}(t_0^{(k)})= \min _{j=1,2,3} g_{I_j}(t_0^{(j)}).$$

For illustration purpose, we shall assume that we have chosen the model parameters such that $k=3$. Thus, the essential index set is given by $I=I_3=\{1,2\}$ and

$$t_0=t_0^{(3)} =\frac{\sqrt{4(\sum _{i\in I} \nu _i\mu _i)^2(1-2H)^2+16 H(1-H) \sum _{i\in I} \nu _i^2 \sum _{i\in I} \mu _i^2 }-2(1-2H)\sum _{i\in I} \nu _i\mu _i}{4\sum _{i\in I} \mu _i^2(1-H)}\in [t'_2, \infty ).$$

We further assume that the model parameters were chosen such that $t_0=t'_2$. In such a case, we have

$$I=\{1,2\}, \ \ K=\{3\}, \ \ J=\{4\}.$$

Next, let us discuss different cases distinguished according to $H \varvec{\nu }_{I} = (1-H) t_0 \varvec{\mu }_{I}$ being valid or not. Following the notation introduced at the beginning of this section, $H \varvec{\nu }_{I} = (1-H) t_0 \varvec{\mu }_{I}$ means that $t_0=t_{0,1}=t_{0,2}$ (see Eq. (13)) and $\zeta _1'(t_0)=\zeta _2'(t_0)=0.$ In contrast, $H \varvec{\nu }_{I} \ne (1-H) t_0 \varvec{\mu }_{I}$ means that $t_0$ falls between $t_{0,1}$ and $t_{0,2}$, and one of $\zeta '_1(t_0)$ and $\zeta '_2(t_0)$ is positive while the other is negative. Consequently, we obtain the exact asymptotics for

$$\mathbb {P} \left( \exists _{t \ge 0} \forall _{i=1 ,\ldots ,4} B_{H,i}(t) -\mu _i t> \nu _iu \right) ,\ \ u\rightarrow \infty ,$$

by applying Theorem 3.1, where

$${\mathcal {C}_K} ={\frac{1}{2\pi t_0^{2H}}} \int _{\mathbb {R}} e^{-\frac{1}{4} g''_I(t_0) y^2} \Phi \left(\frac{\mu _3}{t_0^H} y\right) dy,$$

with $\Phi (\cdot )$ denoting the standard normal distribution function.

## Proof of Theorem 3.1

First note that by self-similarity of the fBms,

$$\begin{aligned} P(u)= & \mathbb {P} \left( \exists _{t \ge 0} \varvec{X}(t) -\varvec{\mu }t> \varvec{\nu }u \right) \\= & \mathbb {P} \left( \exists _{t \ge 0} \varvec{X}(t) > (\varvec{\nu }+\varvec{\mu }t)u^{1-H} \right) . \end{aligned}$$

Hereafter, for simplicity we denote $v=u^{1-H}.$ Furthermore, denote

$$\Delta _v=[t_0- \ln (v)/v, t_0+ \ln (v)/v],\quad \widetilde{\Delta }_v=[0,\infty )\setminus [t_0- \ln (v)/v, t_0+ \ln (v)/v].$$

It follows that

15 $$\begin{aligned} p(v)\le P(u)\le \Pi (v) +p(v), \end{aligned}$$

where

$$p(v)=\mathbb {P} \left( \exists _{t \in \Delta _v} \varvec{X}(t)> (\varvec{\nu }+\varvec{\mu }t) v \right) , \quad \Pi (v)=\mathbb {P} \left( \exists _{t \in \widetilde{\Delta }_v} \varvec{X}(t) > (\varvec{\nu }+\varvec{\mu }t) v \right) .$$

The proof consists of two steps. In Step 1, we obtain the asymptotics of $p(v), v\rightarrow \infty$. In Step 2, we derive a suitable upper bound for $\Pi (v)$ for all large enough *v*, which confirms asymptotic negligibility of $\Pi (v)$ with respect to *p*(*v*) as $v\rightarrow \infty$. The proof is then completed by combining these results. Without loss of generality, we shall only consider the most involved case where $K\ne \emptyset$. Before delving into all the details, for any $M\in (0, \infty ]$ we introduce

16 $$\begin{aligned} {\mathcal {C}_{K,M}}= { \frac{1}{\sqrt{ (2\pi t_0^{2H} )^{\left|I \right|} \left|\Sigma _{II} \right|} } \int _{-M}^M } e^{-\frac{1}{4} g_I''(t_0) y^2} \ \cdot \mathbb {P} \left( \varvec{Y}_K< t_0^{-H} \left(\varvec{\mu }_K - \Sigma _{KI}\Sigma _{II}^{-1} \varvec{\mu }_I\right) y \right) dy. \end{aligned}$$

We note that ${\mathcal {C}_{K}}$, given in Eq. (12) is actually equal to ${\mathcal {C}_{K,\infty }}$.

### Step 1: Analysis of ${ p(v)}$  

The idea is to split the interval $\Delta _v$ into smaller intervals. It turns out that we need to distinguish two different cases (i) and (ii) as stated in Theorem 3.1, for case (i) we shall use intervals of the classical Pickands length, but for case (ii) we need to use intervals of a length that is shorter than the Pickands length. These two cases will be discussed seperately below.

Case (i): $\underline{H<{1}/{2}, \text { or } H>1/2 \text { and } H \varvec{\nu }_{I} = (1-H) t_0 \varvec{\mu }_{I}}.$ Denote, for any fixed $T>0$ and $v>0$

$$\begin{aligned} \triangle _{j;v}=\triangle _{j;v}(T)= [t_0+j Tv^{-1/H}, t_0+(j+1) Tv^{-1/H}],\ \ \ -N_v-1\le j\le N_v, \end{aligned}$$

where $N_v=\lfloor T^{-1} \ln (v) v^{1/H-1}\rfloor$ (we denote by $\lfloor \cdot \rfloor$ the floor function). Also denote $N_{v,M} = \lfloor T^{-1} M v^{1/H-1} \rfloor$ for any $M>0$. By applying the Bonferroni’s inequality, we have

17 $$\begin{aligned} p_1(v)\ge p(v) \ge p_{2,M}(v)-\pi _M(v), \end{aligned}$$

where

$$\begin{aligned} p_1(v)= \sum _{j=-N_v-1}^{N_v}p_{j;v},\ \ p_{2,M}(v) = \sum _{j=-N_{v,M}}^{N_{v,M}}p_{j;v},\ \ \pi _M(v) = \sum _{ -N_{v,M}\le j< l\le N_{v,M}}p_{j,l;v}, \end{aligned}$$

with

$$\begin{aligned} p_{j;v}=\mathbb {P} \left( \exists _{t\in \Delta _{j;v}} \varvec{X}(t)> (\varvec{\alpha }+\varvec{\mu }t)v \right) \end{aligned}$$

and

18 $$\begin{aligned} p_{j,l;v}=\mathbb {P} \left( \exists _{t\in \triangle _{j;v}} \varvec{X}(t)> (\varvec{\alpha }+\varvec{\mu } t)v, \ \exists _{t\in \triangle _{l;v}} \varvec{X}(t)> (\varvec{\alpha }+\varvec{\mu } t) v \right) . \end{aligned}$$

Next, we shall deal with the single-sum $p_1(v)$, $p_{2,M}(v)$ and the double-sum $\pi _M(v)$, respectively. For the asymptotics of the single-sum terms, we shall use the following uniform version of a generalized Pickands lemma. The proof of Lemma 4.1 is displayed in Appendix.

#### Lemma 4.1

Fix $T>0$. We have, as $v\rightarrow \infty ,$

$$\begin{aligned} & \mathbb {P} \left( \exists _{t \in [t_0+\tau v^{-1/H}, t_0+(\tau +T) v^{-1/H}]} \varvec{X}(t) > (\varvec{\nu }+\varvec{\mu }t) v \right) \\ & \sim v^{-\left|I \right|} \mathcal {H}_{I}(T) { \frac{1}{\sqrt{ (2\pi t_0^{2H} )^{\left|I \right|} \left|\Sigma _{II} \right|} }} e^{-\frac{v^2}{2} g_I(t_0+\tau v^{-1/H})}\\ & \quad { \times \mathbb {P} \left( \varvec{Y}_K< -t_0^{-H} \left(\varvec{\mu }_K - \Sigma _{KI}\Sigma _{II}^{-1} \varvec{\mu }_I\right) (\tau v^{{1}-1/H}) \right) }, \end{aligned}$$

holds uniformly in $\tau$ such that $\left|\tau \right|\le T(N_v+1)$, where *K* is the weakly essential index set defined in Eq. (11) and $\varvec{Y}_K$ is given in Eq. (12).

With Lemma 4.1, it is straightforward to check that, as $v\rightarrow \infty ,$

19 $$\begin{aligned} & p_{2,M}(v)\sim { v^{-\left|I \right|+1/H-1} \frac{\mathcal {H}_{I}(T)}{T}\mathcal {C}_{K,M} e^{-\frac{v^2}{2} g_I(t_0)} }, \end{aligned}$$

where ${\mathcal {C}_{K,M}}$ is given in Eq. (16). Indeed, by Lemma 4.1 and Eq. (10), we derive that, as $v\rightarrow \infty ,$

$$\begin{aligned} p_{2,M}(v)\sim & v^{-\left|I \right|+1/H-1} \frac{\mathcal {H}_{I}(T)}{T} { \frac{1}{\sqrt{ (2\pi t_0^{2H} )^{\left|I \right|} \left|\Sigma _{II} \right|} }} e^{-\frac{v^2}{2} g_I(t_0)}\\ & \times \sum _{j=-N_{v,M}}^{N_{v,M}} (T v^{1-1/H}) e^{-\frac{g_I''(t_0)(jT v^{1-1/H})^2}{4}}\\ & \qquad \qquad \quad { \times \mathbb {P} \left( \varvec{Y}_K< - t_0^{-H} \left(\varvec{\mu }_K - \Sigma _{KI}\Sigma _{II}^{-1} \varvec{\mu }_I\right) ({jT} v^{{1}-1/H}) \right) } \end{aligned}$$

Thus, the claim in Eq. (19) follows by letting $v\rightarrow \infty$ and an application of the Lebesgue dominated convergence theorem. Similarly, we have, as $v\rightarrow \infty ,$

20 $$\begin{aligned} p_1(v)\sim { v^{-\left|I \right|+1/H-1} \frac{\mathcal {H}_{I}(T)}{T}\mathcal {C}_K e^{-\frac{v^2}{2} g_I(t_0)} }, \end{aligned}$$

where ${\mathcal {C}_{K}}$ is given in Eq. (12).

Next, we consider the term $\pi _M(v)$, where it is sufficient to assume *T* to be a large number in the sequel. We shall derive a suitable asymptotic upper bound for it, for which we need the following lemma. Denote

$$\begin{aligned} p(\tau _1,\tau _2;v) = \mathbb {P}\Bigg ( \exists \begin{array}{l} t \in [t_0+\tau _1 v^{-1/H},t_0+(\tau _1+1) v^{-1/H}]\\ s \in [t_0+\tau _2 v^{-1/H},t_0+(\tau _2+1) v^{-1/H}]\end{array}: \varvec{X}(t)>(\varvec{\nu }+\varvec{\mu }t) v,\, \varvec{X}(s)>(\varvec{\nu }+\varvec{\mu }s) v \Bigg ). \end{aligned}$$

#### Lemma 4.2

For any fixed $M>0$, there exist $C_M, v_M>0$ such that, for all $v \ge v_M$,

$$\begin{aligned} p(\tau _1,\tau _2;v)\le C_M \exp \left( -C_M^{-1} (\tau _2-\tau _1)^{2H} \right) v^{-|I|} e^{- \frac{v^2 g_I(t_0)}{2}}. \end{aligned}$$

holds uniformly in $\tau _1, \tau _2$ such that $-M v^{1/H-1}\le \tau _1+1\le \tau _2\le M v^{1/H-1}$.

The proof of Lemma 4.2 is displayed in Appendix.

Recall the defination of $p_{j,l;v}$ in Eq. (18). We shall partition the interval $\Delta _{j;v}$ into segments of the form $[(j+1)T-k-1,(j+1)T-k]$, $0 \le k \le \lfloor T\rfloor$ and the interval $\Delta _{l;v}$ into segments of the form $[lT+m,lT+m+1]$, $0\le m \le \lfloor T\rfloor$. By doing so, we have

$$\begin{aligned} p_{j,l;v}\le \sum _{0\le k,m\le \lfloor T\rfloor } p((j+1)T-k-1,lT+m;v). \end{aligned}$$

Applying Lemma 4.2 to the above, we have, for all large enough *v*,

$$\begin{aligned} p_{j,l;v}\le & \sum _{0 \le k,m \le T^{1/3}-1} C_M \exp \left( -C_M^{-1} |(l-j-1)T+m+k+1|^{2H} \right) v^{-|I|} e^{- \frac{v^2 g_I(t_0)}{2}}\\ & + \underset{T^{1/3}-1 \le \max (k,m) \le \lfloor T\rfloor }{\sum _{k,m\ge 0}} C_M \exp \left( -C_M^{-1} |(l-j-1)T+m+k+1|^{2H} \right) v^{-|I|} e^{- \frac{v^2 g_I(t_0)}{2}}\\\le & T^{2/3} C_M \exp \left( -C_M^{-1}( (l-j-1)T)^{2H} \right) v^{-|I|} e^{- \frac{v^2 g_I(t_0)}{2}}\\ & + T^2 C_M \exp \left( -C_M^{-1} ((l-j-1)T+T^{1/3})^{2H} \right) v^{-|I|} e^{- \frac{v^2 g_I(t_0)}{2}}, \end{aligned}$$

uniformly for all *j*, *l* such that $-N_{v,M} \le j < l \le N_{v,M}$. Thus, for all large enough *v*, 

21 $$\begin{aligned} \pi _M(v)\le & \sum _{-N_{v,M} \le j < l \le N_{v,M}} p_{j,l;v}\nonumber \\\le & 2 N_{v,M}T^{2/3} C_M \sum _{l \ge 0} \exp \left( -C_M^{-1}( (lT)^{2H} \right) v^{-|I|} e^{- \frac{v^2 g_I(t_0)}{2}}\\ & +2 N_{v,M} T^2 C_M \sum _{l \ge 0} \exp \left( -C_M^{-1} (lT+T^{1/3})^{2H} \right) v^{-|I|} e^{- \frac{v^2 g_I(t_0)}{2}}. \nonumber \end{aligned}$$

Since for all $x \ge 0$ and $T \ge 1$ we have

$$\exp \left( -C_M^{-1}(lT+x)^{2H}\right) \le \exp \left( -C_M^{-1}(l+x)^{2H}\right) \le \int _{x+l-1}^{x+l} \exp \left( -C_M^{-1}t^{2H}\right) dt,$$

therefore

$$\begin{aligned} \sum _{l \ge 0} \exp \left( -C_M^{-1}(lT+x)^{2H} \right) \le \exp \left( -C_M^{-1}x^{2H} \right) + \int _{x}^{\infty } \exp \left( -C_M^{-1}t^{2H} \right) dt \le \widetilde{C}_M \exp \left( -\widetilde{C}_M^{-1}x^H\right) , \end{aligned}$$

where $\widetilde{C}_M>0$ is a constant independent of *x*. Combining this with Eq. (21), we obtain

22 $$\begin{aligned} \limsup _{T \rightarrow \infty } \lim _{v\rightarrow \infty } \frac{\pi _M(v)}{v^{-|I|+1/H-1}e^{-\frac{v^2 g_I(t_0)}{2}}} = 0. \end{aligned}$$

Applying Eqs. (19), (20) and (22) to Eq. (17), we obtain

23 $$\begin{aligned} { \mathcal {H}_{I}\mathcal {C}_{K,M} }\le & \liminf _{T \rightarrow \infty } \lim _{u\rightarrow \infty }\frac{p(v)}{v^{-|I|+1/H-1}e^{-\frac{v^2 g_I(t_0)}{2}}}\\\le & \limsup _{T \rightarrow \infty } \lim _{u\rightarrow \infty } \frac{p(v)}{v^{-|I|+1/H-1}e^{-\frac{v^2 g_I(t_0)}{2}}} \le { \mathcal {H}_{I}\mathcal {C}_{K} }. \nonumber \end{aligned}$$

Now, letting $M \rightarrow \infty$ in the above, we have

24 $$\begin{aligned} p(v) \sim { v^{-\left|I \right|+1/H-1} \mathcal {H}_{I}\mathcal {C}_{K} e^{-\frac{v^2}{2} g_I(t_0)}}, \ \ v\rightarrow \infty . \end{aligned}$$

Case (ii): $\underline{H>1/2 \text { and } H \varvec{\nu }_{I} \ne (1-H) t_0 \varvec{\mu }_{I}.}$ In this case, we shall consider intervals of length that is shorter than the one used in Case (i). Precisely, we define, for any fixed integer $T>0$ and $v>0$

$$\begin{aligned} \widehat{\Delta }_{j;v}=\widehat{\Delta }_{j;v}(T)= [t_0+j Tv^{-2}, t_0+(j+1) Tv^{-2}],\ \ \ -\widehat{N}_v-1\le j\le \widehat{N}_v, \end{aligned}$$

where $\widehat{N}_v=\lfloor T^{-1} \ln (v) v\rfloor$. Also denote $\widehat{N}_{v,M} = \lfloor T^{-1} M v \rfloor$. By Bonferroni’s inequality we have

25 $$\begin{aligned} \widehat{p}_1(v)\ge p(v) \ge \widehat{p}_{2,M}(v)-\widehat{\pi }_M(v), \end{aligned}$$

where

$$\begin{aligned} \widehat{p}_1(v)= \sum _{j=-\widehat{N}_v-1}^{\widehat{N}_v}\widehat{p}_{j;v},\ \ \widehat{p}_{2,M}(v) = \sum _{j=-\widehat{N}_{v,M}}^{\widehat{N}_{v,M}}\widehat{p}_{j;v},\ \ \widehat{\pi }_M(v) = \sum _{ -\widehat{N}_{v,M}\le j< l\le \widehat{N}_{v,M}}\widehat{p}_{j,l;v}, \end{aligned}$$

with

$$\begin{aligned} \widehat{p}_{j;v}=\mathbb {P} \left( \exists _{t\in \widehat{\Delta }_{j;v}} \varvec{X}(t)> (\varvec{\alpha }+\varvec{\mu } t)v \right) \end{aligned}$$

and

$$\widehat{p}_{j,l;v}=\mathbb {P} \left( \exists _{t\in \widehat{\Delta }_{j;v}} \varvec{X}(t)> (\varvec{\alpha }+\varvec{\mu } t)v, \ \exists _{t\in \widehat{\Delta }_{l;v}} \varvec{X}(t)> (\varvec{\alpha }+\varvec{\mu } t) v \right) .$$

Similarly as in Case (i), we shall deal with the single-sum $\widehat{p}_1(v)$, $\widehat{p}_{2,M}(v)$ and the double-sum $\widehat{\pi }_M(v)$, respectively. For the asymptotics of the single-sum terms, we shall use the following uniform version of a generalized Pickands lemma evaluated on a shorter interval. The proof of Lemma 4.3 is deferred to Appendix.

#### Lemma 4.3

Fix $T>0$. We have, as $v\rightarrow \infty ,$

$$\begin{aligned} & \mathbb {P} \left( \exists _{t \in [t_0+\tau v^{-2}, t_0+(\tau +T) v^{-2}]} \varvec{X}(t) > (\varvec{\nu }+\varvec{\mu }t) v \right) \\ & \sim v^{-\left|I \right|} \frac{ t_0^{2H\left|I \right|}}{ \prod _{i \in I}w_i } \left(1+\frac{T}{t_0^{2H}}\sum _{i \in I} \left( w_i \mu _i - \frac{H}{t_0} w_i b_i\right) _{-} \right) e^{-\frac{v^2}{2} g_I(t_0+\tau v^{-2})}\\ & \quad { \times \mathbb {P} \left( \varvec{Y}_K<- t_0^{-H} \left(\varvec{\mu }_K - \Sigma _{KI}\Sigma _{II}^{-1} \varvec{\mu }_I\right) (\tau v^{-1}) \right) /\sqrt{ (2\pi t_0^{2H} )^{\left|I \right|} }/\sqrt{ \left|\Sigma _{II} \right|} }, \end{aligned}$$

holds uniformly in $\tau$ such that $\left|\tau \right|< T ( \widehat{N}_v+1)$.

With Lemma 4.3 given above, it follows by the same lines of reasoning as in Case (i) that

26 $$\begin{aligned} \begin{aligned} \widehat{p}_{2,M}(v)&\sim v^{-\left|I \right|+1} { \frac{t_0^{2H|I|} \mathcal {C}_{K,M}}{T \prod _{i \in I} w_i} } \left(1+\frac{T}{t_0^{2H}}\sum _{i \in I} \left( w_i \mu _i - \frac{H}{t_0} w_i b_i\right) _{-} \right) e^{-\frac{v^2}{2} g_I(t_0)},\\ \widehat{p}_1(v)&\sim v^{-\left|I \right|+1} { \frac{t_0^{2H|I|} \mathcal {C}_{K}}{T \prod _{i \in I} w_i} } \left(1+\frac{T}{t_0^{2H}}\sum _{i \in I} \left( w_i \mu _i - \frac{H}{t_0} w_i b_i\right) _{-} \right) e^{-\frac{v^2}{2} g_I(t_0)} \end{aligned} \end{aligned}$$

hold, as $$v\rightarrow \infty$$.

Next, in order to derive a suitable upper bound for the double-sum term $\widehat{\pi }_M(u)$, we need an analogue of Lemma 4.2. It is worth noting that Lemma 4.4 below looks similar to Lemma 4.2, but the approach used to prove it is quite different, which is displayed in Appendix.

Denote

$$\begin{aligned} \widehat{p}(\tau _1,\tau _2;v) = \mathbb {P}\Bigg ( \exists \begin{array}{l} t \in [t_0+\tau _1 v^{-2},t_0+(\tau _1+1) v^{-2}]\\ s \in [t_0+\tau _2 v^{-2},t_0+(\tau _2+1) v^{-2}]\end{array}: \varvec{X}(t)>(\varvec{\nu }+\varvec{\mu }t) v,\, \varvec{X}(s)>(\varvec{\nu }+\varvec{\mu }s) v \Bigg ). \end{aligned}$$

#### Lemma 4.4

For any fixed $M>0$, there exist $C_M, v_M>0$ such that, for all $v \ge v_M$,

$$\begin{aligned} \widehat{p}(\tau _1,\tau _2;v)\le C_M \exp \left( -C_M^{-1} (\tau _2-\tau _1) \right) v^{-|I|} e^{- \frac{v^2 g_I(t_0)}{2}} \end{aligned}$$

holds uniformly in $\tau _1, \tau _2$ such that $-M v\le \tau _1+1\le \tau _2\le M v$.

Similarly to Eq. (22), we have from the above lemma that

27 $$\begin{aligned} \limsup _{T \rightarrow \infty } \lim _{v\rightarrow \infty } \frac{\widehat{\pi }_M(v)}{v^{-|I|+1}e^{-\frac{v^2 g_I(t_0)}{2}}} = 0. \end{aligned}$$

Consequently, we conclude from Eqs. (25)-(27) that

28 $$\begin{aligned} p(v) \sim v^{-\left|I \right|+1} { \frac{t_0^{2H(|I|-1)} \mathcal {C}_{K}}{ \prod _{i \in I} w_i} } \left(\sum _{i \in I} \left( w_i \mu _i - \frac{H}{t_0} w_i b_i\right) _{-} \right) e^{-\frac{v^2}{2} g_I(t_0)}, \ \ v\rightarrow \infty . \end{aligned}$$

### Step 2: Analysis of ${\Pi (v)}$  

In order to obtain a sharp upper bound for $\Pi (v)$, we can adapt the same arguments as in Lemma 4.1 of Dȩbicki et al. (2018), which gives that, for sufficiently large *v* it holds that

29 $$\begin{aligned} \begin{aligned} \Pi (v)&=\mathbb {P} \left( \exists _{t \in \widetilde{\Delta }_v} \varvec{X}(t) > (\varvec{\nu }+\varvec{\mu }t) v \right) \\&\le C_0 \exp \left(- \frac{v^2}{2} g_I(t_0)- \frac{\min (g''(t_0+), g''(t_0-))}{C_1} (\ln (v))^2\right), \end{aligned} \end{aligned}$$

where $C_0, C_1>0$ are some constants and $g''(t_0\pm )$ are the one-side second derivatives of *g*(*t*), with $g''(t_0\pm )>0$ that can be confirmed as in the proof of Lemma 2.2. It is noted that generally $g''(t_0+)\ne g''(t_0-)$; see Remark 5.7 of Dȩbicki et al. (2018) for such an example.

In order to complete the proof of Theorem 3.1, we note that by combining Eq. (29) with Eq. (24) or Eq. (18) we get that

$$\Pi (v)=o(p(v)),$$

as $v\rightarrow \infty$. The above, together with Eq. (15) (recalling that $v=u^{1-H}$) completes the proof. $\square$

## Data Availability

Data sharing not applicable to this article as no data sets were generated or analysed during the current study.

## Appendix: Additional proofs

### Proof of Lemma

2.2. The proof follows by the use of similar arguments as in the proof of Lemma 2.2 in Dȩbicki et al. (2018). For completeness we present the main steps of argumentation and only highlight the key differences. First, note that $h(t)= \inf _{\varvec{v} \ge \varvec{\nu }+\varvec{\mu } t} \varvec{v}^\top \Sigma ^{-1} \varvec{v} \in C^1(0,\infty )$ has been proved in Dȩbicki et al. (2018), thus $g\in C^1(0,\infty )$ is established. Next, denote $I(t)\subseteq \{1,2,\ldots , d\}$ to be the index set of the solution to the quadratic programming problem $P_{\Sigma }(\varvec{b}(t))$ for any fixed $t>0$. It follows from Lemma A.4 of Dȩbicki et al. (2018) that $I(t), t>0$ is an almost piecewise constant set function. Namely,

$$I(t)=\sum _{j} I_j\mathbb {I}_{(t\in U_j)},$$

where $U_j$’s are of the following form

$$\begin{aligned} (a,b), [a,b), (a,b], [a,b], \{a\}, (b,\infty ), [b,\infty ), \end{aligned}$$

where $0<a<b< \infty$ and $I_j\subseteq \{1,\ldots ,d\}$. Therefore,

$$\begin{aligned} g(t)=g_{I_j}(t)=\frac{\varvec{\mu }_{I_j}^\top \Sigma ^{-1}_{I_jI_j} \varvec{\mu }_{I_j} t^2 +2\varvec{\nu }_{I_j}^\top \Sigma ^{-1}_{I_jI_j} \varvec{\mu }_{I_j} t +\varvec{\nu }_{I_j}^\top \Sigma ^{-1}_{I_jI_j} \varvec{\nu }_{I_j} }{t^{2H}}, \quad t \in U_j^o, \end{aligned}$$

where $U_j^o$ is the inner set of $U_j$. Furthermore, for any fixed $I_j$, the first derivative of $g_{I_j}$,

$$\begin{aligned} g_{I_j}'(t)= \frac{2 \varvec{\mu }_{I_j}^\top \Sigma ^{-1}_{I_jI_j} \varvec{\mu }_{I_j} (1-H) t^2 +2(1-2H)\varvec{\nu }_{I_j}^\top \Sigma ^{-1}_{I_jI_j} \varvec{\mu }_{I_j} t -2H \varvec{\nu }_{I_j}^\top \Sigma ^{-1}_{I_jI_j} \varvec{\nu }_{I_j} }{t^{2H+1}}. \end{aligned}$$

is negative on the left of the positive root of $g_{I_j}'(t)=0$ and then becomes positive on the right of this root. This means that the function $g_{I_j}(t), t>0$ is decreasing to the left of some point and then becomes increasing. Next, we have $g(t)\rightarrow \infty$ as $t\rightarrow \infty$ or $t\rightarrow 0$. From these and the fact that $g\in C^1(0,\infty )$, and using the same arguments as in Lemma 2.2 of Dȩbicki et al. (2018) we can conclude that the minimizer of the function $g(t), t>0$ is given by $t_0 \in U_j$ for some *j*, which must be of the form Eq. (8) and satisfies Eq. (9). Moreover, elementary calculations show that Eq. (10) is valid, where $g''_I(t_0)>0$ follows from the fact that $2H g_I'(t)+t g_I''(t)>0$ for any non-empty set $I \subset \{1,\dots ,d\}$ and $t>0$. The proof is complete. $\square$

### Proof of Lemma

4.1. It follows that

$$\begin{aligned} & \mathbb {P} \left( \exists _{t \in [t_0+\tau v^{-1/H}, t_0+(\tau +T) v^{-1/H}]} \varvec{X}(t)> (\varvec{\nu }+\varvec{\mu }t) v \right) \\ & = \mathbb {P} \left( \exists _{t \in [0, T]} \varvec{X}(t_0+\tau v^{-1/H}+tv^{-1/H})> (\varvec{\nu }+\varvec{\mu }(t_0+\tau v^{-1/H}+t v^{-1/H})) v \right) \\ & = \mathbb {P} \left( \exists _{t \in [0, T]} \varvec{X}_{v,\tau }(t) > \varvec{b} v + \tau \varvec{\mu }v^{1-1/H}+t \varvec{\mu }v^{1-1/H} \right) , \end{aligned}$$

where $\varvec{X}_{v,\tau }(t) = \varvec{X}(t_0+\tau v^{-1/H}+t v^{-1/H}).$ We shall follow some ideas in the proof of Lemma 4.7 in Dȩbicki et al. (2020). Let $\widetilde{\varvec{b}}$ be the optimal solution of the optimization problem $P_\Sigma (\varvec{b})$. We have

$$\begin{aligned} & \mathbb {P} \left( \exists _{t \in [0, T]} \varvec{X}_{v,\tau }(t)> \varvec{b} v + \tau \varvec{\mu }v^{1-1/H}+t \varvec{\mu }v^{1-1/H} \right) \\ & = \mathbb {P} \left( \exists _{t \in [0, T]} \varvec{X}_{v,\tau }(t) - \widetilde{\varvec{b}}v> (\varvec{b}- \widetilde{\varvec{b}}) v + \tau \varvec{\mu }v^{1-1/H}+t \varvec{\mu }v^{1-1/H} \right) . \end{aligned}$$

Define

$$\varvec{Z}_{v,\tau }(t) :=\overline{\varvec{v}}(\varvec{X}_{v,\tau }(t) - \widetilde{\varvec{b}}v- \tau \varvec{\mu }v^{1-1/H}-t \varvec{\mu }v^{1-1/H})+\varvec{x},$$

where $\overline{\varvec{v}}$ has all components equal to *v* for the indices in *I*, and 1 for the indices in $I^c=K\cup J$. It then follows that

$$\begin{aligned} & \mathbb {P} \left( \exists _{t \in [0, T]} \varvec{X}_{v,\tau }(t) - \widetilde{\varvec{b}}v> (\varvec{b}- \widetilde{\varvec{b}}) v + \tau \varvec{\mu }v^{1-1/H}+t \varvec{\mu }v^{1-1/H} \right) \\ & = v^{-\left|I \right|} \int _{\mathbb {R}^d} \mathbb {P} \left( \exists _{t \in [0, T]} \varvec{Z}_{v,\tau }(t) > (\varvec{b}- \widetilde{\varvec{b}}) \overline{\varvec{v}}v+\varvec{x} \mid \varvec{Z}_{v,\tau }(0)=\varvec{0} \right) \varphi _{\Sigma _{v,\tau }}(\widetilde{\varvec{b}}v+ \tau \varvec{\mu }v^{1-1/H}-\varvec{x}/\overline{\varvec{v}}) d\varvec{x}, \end{aligned}$$

with

$$\Sigma _{v,\tau } := \mathbb {E}\left( \varvec{X}_{v,\tau }(0) \varvec{X}_{v,\tau }(0)^\top \right) = (t_0+\tau v^{-1/H})^{2H} \Sigma .$$

Further, denote

$$\varvec{\chi }_{v,\tau }(t) := (\varvec{Z}_{v,\tau }(t) \mid \varvec{Z}_{v,\tau }(0)=\varvec{0})$$

and

$$r_{v,\tau }(t,s):=\frac{1}{2}\left( (t_0+\tau v^{-1/H}+tv^{-1/H})^{2H} + (t_0+\tau v^{-1/H}+sv^{-1/H})^{2H} -\left|t-s \right|^{2H} v^{-2}\right).$$

We derive that

$$\begin{aligned} \mathbb {E}\left( \varvec{\chi }_{v,\tau }(t) \right)= & \overline{\varvec{v}}\left( \mathbb {E}\left( \varvec{X}_{v,\tau }(t)\mid \varvec{X}_{v,\tau }(0)= \widetilde{\varvec{b}}v+ \tau \varvec{\mu }v^{1-1/H}-\varvec{x}/\overline{\varvec{v}}\right) - \widetilde{\varvec{b}}v- \tau \varvec{\mu }v^{1-1/H}-t \varvec{\mu }v^{1-1/H}\right)+\varvec{x} \\= & \overline{\varvec{v}}\left( r_{v,\tau }(t,0) r_{v,\tau }(0,0)^{-1}( \widetilde{\varvec{b}}v+ \tau \varvec{\mu }v^{1-1/H}-\varvec{x}/\overline{\varvec{v}}) - \widetilde{\varvec{b}}v- \tau \varvec{\mu }v^{1-1/H}-t \varvec{\mu }v^{1-1/H}\right)+\varvec{x} \\= & \left(1- r_{v,\tau }(t,0) r_{v,\tau }(0,0)^{-1}\right) \varvec{x} + \overline{\varvec{v}}\left( ( r_{v,\tau }(t,0) r_{v,\tau }(0,0)^{-1} -1) ( \widetilde{\varvec{b}}v+ \tau \varvec{\mu }v^{1-1/H} ) -t \varvec{\mu }v^{1-1/H}\right). \end{aligned}$$

Next, it follows that

$$\begin{aligned} r_{v,\tau }(t,0) r_{v,\tau }(0,0)^{-1} -1 = \frac{(t_0+\tau v^{-1/H}+tv^{-1/H})^{2H} - (t_0+\tau v^{-1/H})^{2H}}{2 (t_0+\tau v^{-1/H})^{2H}} - \frac{t^{2H} v^{-2}}{2 (t_0+\tau v^{-1/H})^{2H}}, \end{aligned}$$

and

$$\begin{aligned} (t_0+\tau v^{-1/H}+tv^{-1/H})^{2H} - (t_0+\tau v^{-1/H})^{2H} = 2H (t_0+\tau v^{-1/H})^{2H-1} tv^{-1/H} + H(2H-1) (t_0+\tau ' v^{-1/H})^{2H-2} (tv^{-1/H})^2, \end{aligned}$$

with some $\tau ' \in [\tau , \tau +t]$. Some elementary calculations yield that, as $v\rightarrow \infty$,

30 $$\begin{aligned} \mathbb {E}\left( \varvec{\chi }_{v,\tau }(t) \right) \rightarrow \begin{pmatrix} -\frac{1}{2t_0^{2H}}\varvec{b}_I t^{2H} \\ \varvec{0} _{I^c} \end{pmatrix} \end{aligned}$$

holds uniformly for $\tau$ such that $\left|\tau \right|\le T(N_v+1)$, where when $H>1/2$, the condition $H \varvec{\nu }_I = (1-H) t_0 \varvec{\mu }_I$ was used.

Now, we analyze the covariance function of $\varvec{\chi }_{v,\tau }(t), t\ge 0$

$$\begin{aligned} \mathbb {E}\left( [\varvec{\chi }_{v,\tau }(t) -\mathbb {E}\left( \varvec{\chi }_{v,\tau }(t)\right) ][\varvec{\chi }_{v,\tau }(s) -\mathbb {E}\left( \varvec{\chi }_{v,\tau }(s)\right) ]^\top \right) =\text {diag} (\overline{\varvec{v}}) \left( r_{v,\tau }(t,s) -\frac{r_{v,\tau }(t,0) r_{v,\tau }(0,s) }{ r_{v,\tau }(0,0)} \right) \Sigma \ \text {diag} (\overline{\varvec{v}}). \end{aligned}$$

Note that

$$\begin{aligned} & r_{v,\tau }(t,s) r_{v,\tau }(0,0) -r_{v,\tau }(t,0) r_{v,\tau }(0,s) \\ & = ( r_{v,\tau }(t,s) - r_{v,\tau }(t,0) ) r_{v,\tau }(0,0) + r_{v,\tau }(t,0) ( r_{v,\tau }(0,0)- r_{v,\tau }(0,s) )\\ & =\frac{1}{2}\left( (t_0+\tau v^{-1/H}+sv^{-1/H})^{2H} - (t_0+\tau v^{-1/H})^{2H} \right)( r_{v,\tau }(0,0)- r_{v,\tau }(t,0) ) \\ & \ \ \ \ + \frac{1}{2} \left(t^{2H} r_{v,\tau }(0,0) +s^{2H} r_{v,\tau }(t,0) - \left|t-s \right|^{2H} r_{v,\tau }(0,0) \right) v^{-2}. \end{aligned}$$

Thus, similarly to the calculations for the mean, we obtain that, as $v\rightarrow \infty ,$

$$\begin{aligned} \mathbb {E}\left( [\varvec{\chi }_{v,\tau }(t) -\mathbb {E}\left( \varvec{\chi }_{v,\tau }(t)\right) ][\varvec{\chi }_{v,\tau }(s) -\mathbb {E}\left( \varvec{\chi }_{v,\tau }(s)\right) ]^\top \right) \rightarrow \begin{pmatrix} K(t,s)\Sigma _{II} & \varvec{0}_{II^c}\\ \varvec{0}_{I^c I} & \varvec{0}_{I^c I^c} \end{pmatrix} \end{aligned}$$

holds uniformly for $\tau$ such that $\left|\tau \right| \le T(N_v+1)$, where $K(t,s)=\frac{1}{2}(t^{2H}+s^{2H}-\left|t-s \right|^{2H})$. Next, we show that the process $\{\varvec{\chi }_{v,\tau }(t), t\in [0,T]\}$ is tight for all $\tau$ such that $|\tau | \le T(N_v+1)$ and large enough *v*. To this end, it is sufficient to show the tightness of the conditional process $\{\overline{\varvec{v}}\varvec{X}_{v,\tau }(t) \mid \varvec{Z}_{v,\tau }(0)=\varvec{0}, t\in [0,T]\}$. Let us note that for any jointly Gaussian distributed random vectors $\varvec{U}$ and $\varvec{Y}$, it is known that

31 $$\begin{aligned} \mathbb {V}ar\left( \varvec{U} | \varvec{Y} =\varvec{y}\right) \le \mathbb {V}ar\left( \varvec{U}\right) , \end{aligned}$$

where $\mathbb {V}ar\left( \varvec{V}\right) := \mathbb {E}\left( (\varvec{V}-\mathbb {E}\left( \varvec{V}\right) )^{\top }(\varvec{V}-\mathbb {E}\left( \varvec{V}\right) )\right)$ is the sum of variances of the random (column) vector $\varvec{V}$. Hence, for all $t,s\in [0,T],$

$$\begin{aligned} \mathbb {V}ar\left( \overline{\varvec{v}}\varvec{X}_{v,\tau }(t) - \overline{\varvec{v}}\varvec{X}_{v,\tau }(s) \mid \varvec{Z}_{v,\tau }(0)=\varvec{0}\right) \le \mathbb {V}ar\left( \overline{\varvec{v}}\varvec{X}_{v,\tau }(t) - \overline{\varvec{v}}\varvec{X}_{v,\tau }(s)\right) \le C |t-s|^{2H} \end{aligned}$$

holds uniformly for all $\tau$ such that $|\tau | \le T(N_v+1)$ and large enough *v*, where $C>0$ is a constant. Thus, $\{\varvec{\chi }_{v,\tau }(t), t\in [0,T]\}$ is tight.

Therefore, following similar arguments as in Dȩbicki et al. (2020) we conclude that, as $v\rightarrow \infty$,

$$\begin{aligned} \mathbb {P} \left( \exists _{t \in [0, T]} \varvec{Z}_{v,\tau }(t)> (\varvec{b}- \widetilde{\varvec{b}}) \overline{\varvec{v}}v +\varvec{x} \mid \varvec{Z}_{v,\tau }(0)=\varvec{0} \right) \rightarrow \mathbb {P} \left( \exists _{t \in [0, T]} \varvec{W}_{I}(t) - \frac{1}{2t_0^{2H}}\varvec{b}_I t^{2H}>\varvec{x}_I \right) \cdot {\mathbb {I}}_{(\varvec{x}_K < \varvec{0}_K)} \end{aligned}$$

uniformly for all $\tau$ such that $|\tau | \le T(N_v+1)$, where $\varvec{W}_{I}(t) =D \varvec{B}_{H,I}(t)$ with $D D^\top = \Sigma _{II}$.

Next, we have

$$\begin{aligned} & \varphi _{\Sigma _{v,\tau }}(\widetilde{\varvec{b}}v + \tau \varvec{\mu }v^{1-1/H}-\varvec{x}/\overline{\varvec{v}}) =\frac{1}{\sqrt{(2\pi )^d \left|\Sigma _{v,\tau } \right|} }\\ & \ \ \ \ \ \times \exp \left(- \frac{1}{2 (t_0+\tau v^{-1/H})^{2H}} (\widetilde{\varvec{b}}v + \tau \varvec{\mu }v^{1-1/H}-\varvec{x}/\overline{\varvec{v}}) ^\top \Sigma ^{-1} (\widetilde{\varvec{b}}v + \tau \varvec{\mu }v^{1-1/H}-\varvec{x}/\overline{\varvec{v}}) \right)\\ & = \varphi _{\Sigma _{v,\tau }}(\widetilde{\varvec{b}}v + \tau \varvec{\mu }v^{1-1/H}) \exp \left(\frac{1}{ (t_0+\tau v^{-1/H})^{2H}} (\varvec{x}/\overline{\varvec{v}})^\top \Sigma ^{-1} (\widetilde{\varvec{b}}v + \tau \varvec{\mu }v^{1-1/H}) - \frac{1}{2 (t_0+\tau v^{-1/H})^{2H}} (\varvec{x}/\overline{\varvec{v}})^\top \Sigma ^{-1} (\varvec{x}/\overline{\varvec{v}}) \right). \end{aligned}$$

Since $\varvec{w} = \Sigma ^{-1}\widetilde{\varvec{b}}$ and (by Eq. (7)) $\varvec{w}_I =(\Sigma _{II})^{-1} \varvec{b}_I>0$ and $\varvec{w}_{I^c} =\varvec{0}_{I^c}$, the above exponent is asymptotically equal to, as $v\rightarrow \infty ,$

$$\begin{aligned} \frac{1}{t_0^{2H}} \varvec{w}_I^\top \varvec{x}_I +\frac{1}{t_0^{2H}} \varvec{x}_{I^c}^\top (\Sigma ^{-1} \varvec{\mu })_{I^c} ( \tau v^{1-1/H})-\frac{1}{2 t_0^{2H}} \varvec{x}_{I^c}^ \top (\Sigma ^{-1})_{I^cI^c} \varvec{x}_{I^c} \end{aligned}$$

uniformly for $\tau$ such that $\left|\tau \right|\le T(N_v+1)$. Next, we shall rewrite $\varphi _{\Sigma _{v,\tau }}(\widetilde{\varvec{b}}v + \tau \varvec{\mu }v^{1-1/H})$. Note that for any $\varvec{y}\in \mathbb {R}^d$, we have

$$\begin{aligned} (\widetilde{\varvec{b}}+\varvec{y})^\top \Sigma ^{-1} (\widetilde{\varvec{b}}+\varvec{y})= & \varvec{b}_I^\top (\Sigma _{II})^{-1} \varvec{b}_I + 2 \varvec{b}_I^\top (\Sigma _{II})^{-1} \varvec{y}_I +\varvec{y}^\top \Sigma ^{-1} \varvec{y}\\= & (\widetilde{\varvec{b}}+\varvec{y})_I^\top (\Sigma _{II})^{-1} (\widetilde{\varvec{b}}+\varvec{y})_I - \varvec{y}_I^\top (\Sigma _{II})^{-1} \varvec{y}_I +\varvec{y}^\top \Sigma ^{-1} \varvec{y}. \end{aligned}$$

Therefore, as $$v\rightarrow \infty$$,

$$\begin{aligned} \varphi _{\Sigma _{v,\tau }}(\widetilde{\varvec{b}}v + \tau \varvec{\mu }v^{1-1/H})\sim & \frac{1}{ \sqrt{ (2\pi )^d \left|\Sigma _{v,\tau } \right|}}\exp \left(-\frac{v^2}{2} g_I(t_0 +\tau v^{-1/H})\right) \\ & \times \exp \left(- \frac{(\tau v^{1-1/H})^2}{2t_0^{2H}} \left( \varvec{\mu }^\top \Sigma ^{-1} \varvec{\mu }- \varvec{\mu }_I^\top (\Sigma _{II})^{-1} \varvec{\mu }_I \right)\right) \end{aligned}$$

uniformly for $\tau$ such that $\left|\tau \right|\le T(N_v+1)$. Putting everything together and using the dominated convergence theorem as in Dȩbicki et al. (2020) (we omit the standard details), we obtain, as $v\rightarrow \infty$,

32 $$\begin{aligned} & \mathbb {P} \left( \exists _{t \in [t_0+\tau v^{-1/H}, t_0+(\tau +T) v^{-1/H}]} \varvec{X}(t) > (\varvec{\nu }+\varvec{\mu }t) v \right) \nonumber \\ & \sim v^{-\left|I \right|} \frac{\mathcal {H}_{I}(T)}{\sqrt{ (2\pi t_0^{2H})^d \left|\Sigma \right|}} e^{-\frac{v^2}{2} g_I(t_0+\tau v^{-1/H})} \times \exp \left(- \frac{(\tau v^{1-1/H})^2}{2t_0^{2H}} \left( \varvec{\mu }^\top \Sigma ^{-1} \varvec{\mu }- \varvec{\mu }_I^\top (\Sigma _{II})^{-1} \varvec{\mu }_I \right)\right) \nonumber \\ & \ \ \times \int _{\mathbb {R}^{\left|I^c \right|}} e^{\frac{1}{t_0^{2H}} \varvec{x}_{I^c}^\top (\Sigma ^{-1} \varvec{\mu })_{I^c} ( \tau v^{1-1/H})-\frac{1}{2 t_0^{2H}} \varvec{x}_{I^c}^ \top (\Sigma ^{-1})_{I^cI^c} \varvec{x}_{I^c}} {\mathbb {I}}_{(\varvec{x}_K < \varvec{0}_K)} d\varvec{x}_{I^c} \end{aligned}$$

uniformly for $\tau$ such that $\left|\tau \right|\le T(N_v+1)$. Furthermore, using the Schur complement of invertible block matrix and some elementary calculations, it can be derived that

$$\begin{aligned} \varvec{\mu }^\top \Sigma ^{-1} \varvec{\mu }- \varvec{\mu }_I^\top (\Sigma _{II})^{-1} \varvec{\mu }_I = (\Sigma ^{-1} \varvec{\mu })_{I^c}^\top [(\Sigma ^{-1})_{I^cI^c}]^{-1} (\Sigma ^{-1} \varvec{\mu })_{I^c}. \end{aligned}$$

Therefore,

$$\begin{aligned} & - \frac{(\tau v^{1-1/H})^2}{2t_0^{2H}} \left( \varvec{\mu }^\top \Sigma ^{-1} \varvec{\mu }- \varvec{\mu }_I^\top (\Sigma _{II})^{-1} \varvec{\mu }_I \right)+\frac{1}{t_0^{2H}} \varvec{x}_{I^c}^\top (\Sigma ^{-1} \varvec{\mu })_{I^c} ( \tau v^{1-1/H})-\frac{1}{2 t_0^{2H}} \varvec{x}_{I^c}^ \top (\Sigma ^{-1})_{I^cI^c} \varvec{x}_{I^c}\\ & =-\frac{1}{2t_0^{2H}}[\varvec{x}_{I^c} - ( \tau v^{1-1/H}) [(\Sigma ^{-1})_{I^cI^c}]^{-1} (\Sigma ^{-1} \varvec{\mu })_{I^c}]^\top (\Sigma ^{-1})_{I^cI^c} [\varvec{x}_{I^c} - ( \tau v^{1-1/H}) [(\Sigma ^{-1})_{I^cI^c}]^{-1} (\Sigma ^{-1} \varvec{\mu })_{I^c}]. \end{aligned}$$

Hence

$$\begin{aligned}&\exp \left(- \frac{(\tau v^{1-1/H})^2}{2t_0^{2H}} \left( \varvec{\mu }^\top \Sigma ^{-1} \varvec{\mu }- \varvec{\mu }_I^\top (\Sigma _{II})^{-1} \varvec{\mu }_I \right)\right) \times \int _{\mathbb {R}^{\left|I^c \right|}} e^{\frac{1}{t_0^{2H}} \varvec{x}_{I^c}^\top (\Sigma ^{-1} \varvec{\mu })_{I^c} ( \tau v^{1-1/H})-\frac{1}{2 t_0^{2H}} \varvec{x}_{I^c}^ \top (\Sigma ^{-1})_{I^cI^c} \varvec{x}_{I^c}} {\mathbb {I}}_{(\varvec{x}_K< \varvec{0}_K)} d\varvec{x}_{I^c}\\&\qquad = \int _{\mathbb {R}^{\left|I^c \right|}} e^{-\frac{1}{2t_0^{2H}}[\varvec{x}_{I^c} - ( \tau v^{1-1/H}) [(\Sigma ^{-1})_{I^cI^c}]^{-1} (\Sigma ^{-1} \varvec{\mu })_{I^c}]^\top (\Sigma ^{-1})_{I^cI^c} [\varvec{x}_{I^c} - ( \tau v^{1-1/H}) [(\Sigma ^{-1})_{I^cI^c}]^{-1} (\Sigma ^{-1} \varvec{\mu })_{I^c}]} {\mathbb {I}}_{(\varvec{x}_K< \varvec{0}_K )} d\varvec{x}_{I^c}\\&\qquad = \int _{\mathbb {R}^{\left|I^c \right|}} e^{-\frac{1}{2 t_0^{2H}} \varvec{x}_{I^c}^ \top (\Sigma ^{-1})_{I^cI^c} \varvec{x}_{I^c}} {\mathbb {I}}_{(\varvec{x}_K < - ( \tau v^{1-1/H}) [[(\Sigma ^{-1})_{I^cI^c}]^{-1} (\Sigma ^{-1} \varvec{\mu })_{I^c}]_K)} d\varvec{x}_{I^c}. \end{aligned}$$

On the other hand, we have, from the Schur complement formula,

$$\begin{aligned} & [(\Sigma ^{-1})_{I^cI^c}]^{-1}= \Sigma _{I^c I^c} - \Sigma _{I^c I}\Sigma _{II}^{-1} \Sigma _{I I^c}, \\ & (\Sigma ^{-1})_{I^cI}=- (\Sigma ^{-1})_{I^cI^c} \Sigma _{I^c I}\Sigma _{II}^{-1}. \end{aligned}$$

This implies that

33 $$\begin{aligned} & \int _{\mathbb {R}^{\left|I^c \right|}} e^{-\frac{1}{2 t_0^{2H}} \varvec{x}_{I^c}^ \top (\Sigma ^{-1})_{I^cI^c} \varvec{x}_{I^c}} {\mathbb {I}}_{(\varvec{x}_K< - ( \tau v^{1-1/H}) [[(\Sigma ^{-1})_{I^cI^c}]^{-1} (\Sigma ^{-1} \varvec{\mu })_{I^c}]_K)} d\varvec{x}_{I^c} \nonumber \\ & = \sqrt{\frac{(2\pi t_0^{2H})^{|I^c|}}{ |(\Sigma ^{-1})_{I^c I^c}| } } \ \mathbb {P} \left( \varvec{Z}_{K}<- ( \tau v^{1-1/H}) t_0^{-H} \left(\varvec{\mu }_{I^c} - \Sigma _{I^c I}\Sigma _{II}^{-1} \varvec{\mu }_I\right)_K, \varvec{Z}_{I^c \setminus K}<\varvec{\infty }_{I^c \setminus K} \right) \nonumber \\ & = \sqrt{\frac{(2\pi t_0^{2H})^{|I^c|}}{ |(\Sigma ^{-1})_{I^c I^c}| } } \ \mathbb {P} \left( \varvec{Y}_K< - ( \tau v^{1-1/H}) t_0^{-H} \left(\varvec{\mu }_{K} - \Sigma _{K I}\Sigma _{II}^{-1} \varvec{\mu }_I \right) \right) , \end{aligned}$$

where

$$\varvec{Z}_{I^c} \overset{d}{\sim }\mathcal {N}\left(\varvec{0}_{I^c}, \Sigma _{I^c I^c} - \Sigma _{I^c I}\Sigma _{II}^{-1} \Sigma _{I I^c} \right),\ \ \varvec{Z}_K \overset{d}{\sim }\varvec{Y}_K \overset{d}{\sim }\mathcal {N}\left(\varvec{0}_K, \Sigma _{K K} - \Sigma _{K I}\Sigma _{II}^{-1} \Sigma _{I K} \right).$$

Consequently, the required result is obtained by noting that $|\Sigma ||(\Sigma ^{-1})_{I^c I^c}|=|\Sigma _{II}|$ (which follows from the Schur complement formula). $\square$

### Proof of Lemma

4.2 Hereafter the exact values of the constants $C_M,v_M,C_{M,\epsilon }$ and $v_{M,\epsilon }$ are not important and can be changed from line to line, where $\epsilon >0$ is a small enough constant which may also change from line to line. Moreover, all the inequalities hold uniformly in $\tau _1,\tau _2 \in [-Mv^{1/H-1}-1,Mv^{1/H-1}]$ such that $\tau _2 -\tau _1 \ge 1$ for large enough *v*, and the constants do not depend on $\tau _1,\tau _2$.

Without loss of generality, we shall assume that $I = \{1,\ldots , d\},$ since otherwise, an upper bound for $p(\tau _1, \tau _2; v)$ with only *I* components can be used. For convenience of notation we sometimes also keep index *I* though it can be omitted or replaced by $\{1,\ldots , d\}$. Denote

$$\begin{aligned} \widetilde{\varvec{X}}_v(t,s):= \frac{1}{2}(\varvec{X}_v(t)+\varvec{X}_v(s)), \ \ \ \text {with}\ \varvec{X}_v(t):= \frac{\varvec{X}(t_0+tv^{-1/H})}{(t_0+tv^{-1/H})^H} \end{aligned}$$

and

$$\begin{aligned} \varvec{b}_v(t_1,t_2) := \frac{1}{2} \left( \varvec{\zeta }(t_0+t_1v^{-1/H}) + \varvec{\zeta }(t_0+t_2v^{-1/H}) \right) , \ \ \ \text {with}\ \varvec{\zeta }(t):= \frac{\varvec{\mu }+\varvec{\nu }t}{t^H}. \end{aligned}$$

It follows that

34 $$\begin{aligned} p(\tau _1,\tau _2;v) \le \mathbb {P} \left( \exists t_1 \in [\tau _1,\tau _1+1],t_2 \in [\tau _2,\tau _2+1]:\widetilde{\varvec{X}}_v(t_1,t_2)>v\varvec{b}_v(t_1,t_2) \right) . \end{aligned}$$

Next, let

$$\begin{aligned} r_v(t,s) := \mathbb {E}\left( \frac{B_{H,1}(t_0+tv^{-1/H})}{(t_0+tv^{-1/H})^H} \frac{B_{H,1}(t_0+sv^{-1/H})}{(t_0+sv^{-1/H})^H} \right) = \frac{(t_0+tv^{-1/H})^{2H}+(t_0+sv^{-1/H})^{2H}-|t-s|^{2H} v^{-2}}{2(t_0+tv^{-1/H})^{H}(t_0+sv^{-1/H})^{H}}. \end{aligned}$$

We have, for $t_1,s_1 \in [\tau _1,\tau _1+1],t_2,s_2 \in [\tau _2,\tau _2+1]$,

$$\begin{aligned} \widetilde{R}_v(t_1,t_2,s_1,s_2):= & \mathbb {E}\left( \widetilde{\varvec{X}}_v(t_1,t_2)\widetilde{\varvec{X}}^{\top }_v(s_1,s_2)\right) \\= & \frac{1}{4} \left( R_v(t_1,s_1)+R_v(t_1,s_2)+R_v(t_2,s_1)+R_v(t_2,s_2)\right) \\= & \widetilde{r}_v(t_1,t_2,s_1,s_2) \Sigma , \end{aligned}$$

where

$$\begin{aligned} & R_v(t,s) := \mathbb {E}\left( \varvec{X}_v(t)\varvec{X}^{\top }_v(s)\right) =r_v(t,s)\Sigma ,\\ & \widetilde{r}_v(t_1,t_2,s_1,s_2):=\frac{1}{4} \left( r_v(t_1,s_1)+r_v(t_1,s_2)+r_v(t_2,s_1)+r_v(t_2,s_2)\right) . \end{aligned}$$

Moreover, we denote

$$\begin{aligned} \widetilde{\Sigma }_v(t_1,t_2) := \widetilde{R}(t_1,t_2,t_1,t_2), \ \ \widetilde{\varvec{w}}_v(\tau _1,\tau _2) := \widetilde{\Sigma }_v^{-1}(\tau _1,\tau _2)\varvec{b}_v(\tau _1,\tau _2). \end{aligned}$$

By conditioning on $\widetilde{\varvec{X}}_v(\tau _1,\tau _2) = v\varvec{b}_v(\tau _1,\tau _2)+\varvec{x}/v$ and using the law of total probability, we obtain, continuing Eq. (34),

35 $$\begin{aligned} & p(\tau _1,\tau _2;v)\nonumber \\ & \le v^{-\left|I \right|} \int _{\mathbb {R}^{\left|I \right|}} \varphi _{\widetilde{\Sigma }_v(\tau _1,\tau _2)}\left( v\varvec{b}_v(\tau _1,\tau _2)-\frac{\varvec{x}}{v}\right) \mathbb {P} \left( \exists t_1 \in [\tau _1,\tau _1+1],t_2 \in [\tau _2,\tau _2+1]:\varvec{\chi }_v(t_1,t_2)>\varvec{x} \right) d\varvec{x} \nonumber \\ & \le v^{-\left|I \right|} \varphi _{\widetilde{\Sigma }_v(\tau _1,\tau _2)}\left( v\varvec{b}_v(\tau _1,\tau _2)\right) \int _{\mathbb {R}^{\left|I \right|}} e^{(\widetilde{\varvec{w}}_v(\tau _1,\tau _2))^{\top } \varvec{x}} \mathbb {P} \left( \exists t_1 \in [\tau _1,\tau _1+1],t_2 \in [\tau _2,\tau _2+1]:\varvec{\chi }_{v}(t_1,t_2)>\varvec{x} \right) d\varvec{x}, \end{aligned}$$

where

$$\varvec{\chi }_v(t_1,t_2) := v\left( \widetilde{\varvec{X}}_v(t_1,t_2) - v\varvec{b}_v(t_1,t_2)\right) +\varvec{x}\Big {|} \left( v\left( \widetilde{\varvec{X}}_v(\tau _1,\tau _2) - v\varvec{b}_v(\tau _1,\tau _2)\right) +\varvec{x}=0 \right) ,$$

and in the last inequality we used the inequality

$$\varphi _{\widetilde{\Sigma }_v(\tau _1,\tau _2)}\left( v\varvec{b}_v(\tau _1,\tau _2)-\frac{\varvec{x}}{v}\right) \le \varphi _{\widetilde{\Sigma }_v(\tau _1,\tau _2)}\left( v\varvec{b}_v(\tau _1,\tau _2)\right) \cdot e^{(\widetilde{\varvec{w}}_v(\tau _1,\tau _2))^{\top } \varvec{x}}.$$

In the following, we shall derive suitable bounds for the integral and the term $\varphi _{\widetilde{\Sigma }_v(\tau _1,\tau _2)}\left( v\varvec{b}_v(\tau _1,\tau _2)\right)$ in Eq. (35), respectively. We start with the integral term, for which we shall apply (Ievlev 2024, Lemma 8).

First, note that for any small $\epsilon >0$ there exists $C_{M,\epsilon }>0$ and $v_{M,\epsilon }>0$ such that for all $v \ge v_{M,\epsilon }$ and all $t_1,s_1 \in [\tau _1,\tau _1 +1]$ and $t_2,s_2 \in [\tau _2,\tau _2+1]$ it holds that

36 $$\begin{aligned} v^2 \left| \widetilde{r}_v(t_1,t_2,s_1,s_2)-\left( 1-\frac{(\tau _2-\tau _1)^{2H}v^{-2}}{4t_0^{2H}}\right) \right|\le & C_{M,\epsilon } +\epsilon (\tau _2-\tau _1)^{2H}. \end{aligned}$$

Indeed, by the Taylor’s formula and the inequalities $|a^{2H}-b^{2H}| \le |a-b|^{2H}$ for $a,b>0,H \in (0,1/2)$ and $|a^{2H}-b^{2H}| \le 2H\max (a,b)^{2H-1} |a-b| \le 2H( \epsilon \max (a,b)^{2H}+\epsilon ^{-2H+1}|a-b|^{2H})$ for $a,b>0, H \in (1/2,1)$, we derive that

37 $$\begin{aligned} v^2 \left| r_v(t_i,s_j)-\left( 1-\frac{|\tau _i-\tau _j|^{2H}v^{-2}}{2t_0^{2H}}\right) \right|\le & C_{M,\epsilon } (|t_i-s_j|^2 v^{2-2/H})+ \left| \frac{|t_i-s_j|^{2H} }{2|t_0+t_i v^{-1/H}|^{H}|t_0+s_i v^{-1/H}|^{H}} - \frac{|\tau _i-\tau _j|^{2H}}{2 t_0^{2H}} \right| \nonumber \\\le & C_{M,\epsilon }(1+\left| |t_i-s_j|^{2H}-|\tau _i-\tau _j|^{2H}\right| )+\epsilon |\tau _i-\tau _j|^{2H} \nonumber \\\le & C_{M,\epsilon }+\epsilon |\tau _i-\tau _j|^{2H} \end{aligned}$$

holds for any $i,j \in \{1, 2\}$. Thus, by summing the inequalities Eq. (37) over $1 \le i,j \le 2$, we establish Eq. (36).

Next, from Eq. (36) it directly follows that there exists $C_{M,\epsilon }>0$ and $v_{M,\epsilon }>0$ such that for all $v \ge v_{M,\epsilon }$ and all $t_1,s_1, \widetilde{t}_1,\widetilde{s}_1 \in [\tau _1,\tau _1 +1]$ and $t_2,s_2, \widetilde{t}_2,\widetilde{s}_2 \in [\tau _2,\tau _2+1]$,

38 $$\begin{aligned} v^2 \left| \widetilde{r}_v(t_1,t_2,s_1,s_2)-\widetilde{r}_v(\widetilde{t}_1,\widetilde{t}_2, \widetilde{s}_1,\widetilde{s}_2)\right| \le C_{M,\epsilon } +\epsilon (\tau _2-\tau _1)^{2H}. \end{aligned}$$

Additionally, there exists $C_M>0$ and $v_M>0$ such that for all $v \ge v_M$ and all $t,s \in [\tau _k,\tau _k+1]$ with $k \in \{1,2\}$,

39 $$\begin{aligned} v^2(1-r_v(t,s))\le C_M|t-s|^{2H}. \end{aligned}$$

Note that, as $v\rightarrow \infty$, $\varvec{\widetilde{w}}_v(\tau _1, \tau _2) \rightarrow \varvec{w}/ t_0^{H} >\varvec{0}.$ Thus,

40 $$\begin{aligned} \varvec{\widetilde{w}}_v(\tau _1, \tau _2) \le \frac{2}{t_0^{H}} \varvec{w} =: \overline{ \varvec{w}} \end{aligned}$$

holds for all large *v*.

In order to get a proper upper bound for the integral term in Eq. (35), we shall apply (Ievlev 2024, Lemma 8), for which we check the following three conditions (recall that $F\subseteq I=\{1,\ldots ,d\}$ is defined such that $\varvec{x}_F>\varvec{0}$ and $\varvec{x}_{I\setminus F}<\varvec{0}$ in the integral in Eq. (35)):

41 $$\begin{aligned} & \sup _{F\subseteq I} \sup _{t,s\in [0,1]} \overline{\varvec{w}}_F ^\top \mathbb {E}\left( \varvec{\chi }_{v,F}(\tau _1+t,\tau _2+s)\right) \le C_{M,\epsilon }+ \epsilon (\tau _2-\tau _1)^{2H}+\epsilon \sum _{j=1}^d \left|x_j \right|,\end{aligned}$$

42 $$\begin{aligned} & \sup _{F\subseteq I} \sup _{t,s\in [0,1]} \text {Var} \left(\overline{\varvec{w}}_F ^\top \varvec{\chi }_{v,F}(\tau _1+t,\tau _2+s)\right) \le C_{M,\epsilon }+\epsilon (\tau _2-\tau _1)^{2H}, \end{aligned}$$

and, for any $F\subseteq I$ and $t_1,s_1 \in [\tau _1,\tau _1+1]$, $t_2,s_2 \in [\tau _2,\tau _2 + 1]$

43 $$\begin{aligned} \text {Var} \left( \overline{\varvec{w}}_F^\top \varvec{\chi }_{v,F}(t_1,t_2) -\overline{\varvec{w}}_F^\top \varvec{\chi }_{v,F}(s_1,s_2)\right)\le C_M\left( (|t_1-s_1|^{2H}+|t_2-s_2|^{2H} \right) \end{aligned}$$

hold for all large enough *v*.

Inequality  (41). Note that by Lagrange’s mean value theorem there exist $C_M>0$, $v_M>0$ such that for $v \ge v_M$ and $t_1 \in [\tau _1,\tau _1+1]$ and $t_2 \in [\tau _2,\tau _2+1]$ it holds that, if $H < {1}/{2}$, then

$$v^2 \biggl |\varvec{b}_v(t_1,t_2)-\varvec{b}_v(\tau _1,\tau _2) \biggr | \le v^{2-1/H} \sup _{s \in [\tau _1,\tau _1+1] \cup [\tau _2,\tau _2+1]} \biggl |\varvec{\zeta }'_I(t_0+sv^{-1/H}) \biggr | \le C_M,$$

and, if $H>1/2$ and $H \varvec{\nu }_I = (1-H) t_0 \varvec{\mu }_I$ (i.e., $\varvec{\zeta }'_I(t_0)=0$), then, by Taylor’s formula with Lagrange’s remainder,

$$\begin{aligned} v^2 \biggl |\varvec{b}_v(t_1,t_2)-\varvec{b}_v(\tau _1,\tau _2) \biggr |\le & v^2 \biggl |\varvec{b}_v(t_1,t_2)-\varvec{\zeta }_I(t_0) \biggr |+v^2 \biggl |\varvec{b}_v(\tau _1,\tau _2)-\varvec{\zeta }_I(t_0) \biggr |\\\le & v^{2-2/H} (|\tau _1|+|\tau _2|+2)^2 \sup _{|s| \le \max (|\tau _1|,|\tau _2|)+1} |\varvec{\zeta }''_I(t_0+sv^{-1/H})|\\\le & C_M. \end{aligned}$$

Hence, for any small $\epsilon >0$ there exist $C_{M,\epsilon },v_{M,\epsilon }>0$ such that for all $v \ge v_{M,\epsilon }$ and all $t_1 \in [\tau _1,\tau _1+1], t_2 \in [\tau _2,\tau _2+1]$,

$$\begin{aligned} & \left| \mathbb {E}\left( \varvec{\chi }_v(t_1,t_2)\right) \right| = \left| \left( -v^2 \varvec{b}_v(t_1,t_2) + \varvec{x}\right) - \widetilde{R}_v(t_1,t_2,\tau _1,\tau _2)\widetilde{\Sigma }_v^{-1}(\tau _1,\tau _2) \left( -v^2 \varvec{b}_v(\tau _1,\tau _2)+\varvec{x}\right) \right| \nonumber \\ & \ \ \le v^2 \left| \left( \widetilde{\Sigma }_v(\tau _1,\tau _2)-\widetilde{R}_v(t_1,t_2,\tau _1,\tau _2)\right) \widetilde{\Sigma }_v^{-1}(\tau _1,\tau _2) \varvec{b}_v(\tau _1,\tau _2) \right| +C_{M,\epsilon }+\epsilon \left| \varvec{x}\right| \nonumber \\ & \ \ \le C_{M,\epsilon } v^2\left| \left( \widetilde{r}_v(\tau _1,\tau _2,\tau _1,\tau _2)-\widetilde{r}_v(t_1,t_2,\tau _1,\tau _2)\right) \right| + C_{M,\epsilon }+\epsilon \left| \varvec{x}\right| \nonumber \\ & \ \ \le C_{M,\epsilon }+ \epsilon ((\tau _2-\tau _1)^{2H}+\left| \varvec{x}\right| ), \end{aligned}$$

where the second inequality follows by the fact that $\lim _{v\rightarrow \infty } \widetilde{r}_v^{-1}(\tau _1,\tau _2,\tau _1,\tau _2) \varvec{b}_v(\tau _1,\tau _2) = \varvec{b}/ t_0^{H}$ and the third (last) inequality follows by using Eq. (38). This yields Eq. (41).

Inequality (42). Note that, for any $t_1 \in [\tau _1,\tau _1+1], t_2 \in [\tau _2,\tau _2+1]$,

$$\begin{aligned} \textrm{Var}(\overline{\varvec{w}}_F^{\top } \varvec{\chi }_{v,F}(t_1,t_2)) = \overline{\varvec{w}}_F^{\top } \Sigma _{FF} \overline{\varvec{w}}_F \cdot K_v(t_1,t_2,t_1,t_2), \end{aligned}$$

where $$K_v(t_1,t_2,s_1,s_2) := v^2$$$$\left( \widetilde{r}_v(t_1,t_2,s_1,s_2)-\widetilde{r}_v(t_1,t_2,\tau _1,\tau _2) \widetilde{r}_v^{-1}(\tau _1,\tau _2,\tau _1,\tau _2)\widetilde{r}_v(\tau _1,\tau _2,s_1,s_2) \right)$$. It follows from Eq. (38) that, for any small $$\epsilon >0$$ there exist $$C_{M,\epsilon },v_{M,\epsilon }>0$$ such that for all $$v \ge v_{M,\epsilon }$$ and all $$t_1,s_1 \in [\tau _1,\tau _1+1], t_2,s_2 \in [\tau _2,\tau _2+1]$$,

$$\begin{aligned} |K_v(t_1,t_2,s_1,s_2)|\le & C_{M,\epsilon }v^2 \biggl |\widetilde{r}_v(t_1,t_2,s_1,s_2)\widetilde{r}_v(\tau _1,\tau _2,\tau _1,\tau _2)-\widetilde{r}_v(t_1,t_2,\tau _1,\tau _2)\widetilde{r}_v(\tau _1,\tau _2,s_1,s_2) \biggr | \nonumber \\\le & C_{M,\epsilon }+\epsilon |\tau _1-\tau _2|^{2H}. \end{aligned}$$

This implies Eq. (42).

Inequality (43). We have, using Eq. (31), that for $t_1,s_1 \in [\tau _1,\tau _1+1]$, $t_2,s_2 \in [\tau _2,\tau _2 + 1]$,

$$\begin{aligned} \text {Var}\left( \overline{\varvec{w}}_F^{\top } \varvec{\chi }_{v,F}(t_1,t_2)-\overline{\varvec{w}}_F^{\top } \varvec{\chi }_{v,F}(s_1,s_2)\right) \le v^2 \text {Var}\left( \overline{\varvec{w}}_F^{\top } \widetilde{\varvec{X}}_{v,F}(t_1,t_2)-\overline{\varvec{w}}_F^{\top } \widetilde{\varvec{X}}_{v,F}(s_1,s_2)\right) . \end{aligned}$$

Furthermore, there exist $C_M>0$, $v_M>0$ such that for all $v \ge v_M$ and all $t_1,s_1 \in [\tau _1,\tau _1+1]$, $t_2,s_2 \in [\tau _2,\tau _2 + 1]$,

44 $$\begin{aligned} & v^2 \text {Var}\left( \overline{\varvec{w}}_F^{\top } \widetilde{\varvec{X}}_{v,F}(t_1,t_2)-\overline{\varvec{w}}_F^{\top } \widetilde{\varvec{X}}_{v,F}(s_1,s_2)\right) \nonumber \\ & \le \frac{v^2}{2}\textrm{Var}\left( \overline{\varvec{w}}^{\top }_F \widetilde{\varvec{X}}_{v,F}(t_1)-\overline{\varvec{w}}^{\top }_F \widetilde{\varvec{X}}_{v,F}(s_1) \right) + \frac{v^2}{2}\textrm{Var}\left( \overline{\varvec{w}}^{\top }_F \widetilde{\varvec{X}}_{v,F}(t_2)-\overline{\varvec{w}}^{\top }_F \widetilde{\varvec{X}}_{v,F}(s_2) \right) \nonumber \\ & \le C_M (|t_1-s_1|^{2H}+|t_2-s_2|^{2H}), \end{aligned}$$

where the last inequality follows from Eq. (39). Thus, Eq. (43) is established.

Consequently, an application of (Ievlev (2024), Lemma 8) yields that, for any small $\epsilon >0$ there exist $C_{M,\epsilon },v_{M,\epsilon }>0$ such that, for all $v \ge v_{M,\epsilon }$,

45 $$\begin{aligned} \int _{\mathbb {R}^{\left|I \right|}} e^{(\widetilde{\varvec{w}}_v(\tau _1,\tau _2))^{\top } \varvec{x}} \mathbb {P} \left( \exists t_1 \in [\tau _1,\tau _1+1],t_2 \in [\tau _2,\tau _2+1]:\varvec{\chi }_{v}(t_1,t_2)>\varvec{x} \right) d\varvec{x} \le e^{C_{M,\epsilon }+\epsilon (\tau _2-\tau _1)^{2H}}. \end{aligned}$$

It remains to estimate $\varphi _{\widetilde{\Sigma }_v(\tau _1,\tau _2)}\left( v\varvec{b}_v(\tau _1,\tau _2)\right)$. By Taylor’s formula with Lagrange’s remainder we derive that there exist $C_{M},v_{M}>0$ such that, for all $v \ge v_{M}$,

$$\begin{aligned} v^2 \biggl |\varvec{b}_v(\tau _1,\tau _2)-\varvec{\zeta }_I\left( t_0+\frac{\tau _1+\tau _2}{2}v^{-1/H}\right) \biggr | \le v^{2-2/H}|\tau _1-\tau _2|^{2} \sup _{s \in [\tau _1,\tau _2]} |\varvec{\zeta }''_I(t_0+sv^{-1/H})| \le C_M. \end{aligned}$$

Hence, there exists $C>0$ such that for any small $\epsilon >0$ there exist $C_{M,\epsilon }, C_{M}, v_{M,\epsilon }>0$ such that, for all $v \ge v_{M,\epsilon }$,

$$\begin{aligned} \varphi _{\widetilde{\Sigma }_v(\tau _1,\tau _2)}\left( v\varvec{b}_v(\tau _1,\tau _2)\right)\le & C_{M,\epsilon }\exp \left( -\frac{v^2}{2} \varvec{b}_v^{\top }(\tau _1,\tau _2) \widetilde{\Sigma }_v^{-1}(\tau _1,\tau _2) \varvec{b}_v(\tau _1,\tau _2) \right) \\= & C_{M,\epsilon }\exp \left( -\frac{v^2}{2}\widetilde{r}_v^{-1}(\tau _1,\tau _2,\tau _1,\tau _2) \varvec{b}_v^{\top }(\tau _1,\tau _2) \Sigma _{II}^{-1}\varvec{b}_v(\tau _1,\tau _2) \right) \\\le & C_{M,\epsilon }\exp \left( -\frac{v^2}{2}\widetilde{r}_v^{-1}(\tau _1,\tau _2,\tau _1,\tau _2) g_I\left( t_0+\frac{\tau _1+\tau _2}{2}v^{-1/H} \right) \right) \\\le & C_{M,\epsilon } \exp \left( -\left( \frac{v^2}{2} +\left( \frac{1}{4t_0^{2H}}-\epsilon \right) (\tau _2-\tau _1)^{2H} \right) g_I\left( t_0+\frac{\tau _1+\tau _2}{2}v^{-1/H} \right) \right) \\\le & C_{M,\epsilon } \exp \left( -\frac{v^2}{2} g_I\left( t_0\right) -C_{M}(\tau _2-\tau _1)^{2H} \right) , \end{aligned}$$

where the penultimate inequality follows from Eq. (36). This, together with Eqs. (35) and (45), establishes the claim of Lemma 4.2. $\square$

### Proof of Lemma

4.3 The proof is analogous to the proof of Lemma 4.1, and thus we shall only present the main differences and some key calculations. We define

$$\begin{aligned} & \varvec{X}_{v,\tau }(t) := \varvec{X}(t_0+\tau v^{-2} + t v^{-2}), \ \ \Sigma _{v,\tau } = \mathbb {E}\left( \varvec{X}_{v,\tau }(0) \varvec{X}_{v,\tau }(0)^{\top }\right) =(t_0+\tau v^{-2})^{2H}\Sigma ,\\ & \varvec{Z}_{v,\tau }(t) := \overline{\varvec{v}}\left( \varvec{X}_{v,\tau }(t)-\widetilde{\varvec{b}}v-\tau \varvec{\mu } v^{-1}-t\varvec{\mu }v^{-1} \right) +\varvec{x},\\ & r_{v,\tau }(t,s):=\frac{1}{2}\left( (t_0+\tau v^{-2}+tv^{-2})^{2H} + (t_0+\tau v^{-2}+sv^{-2})^{2H} -\left|t-s \right|^{2H} v^{-4H}\right). \end{aligned}$$

Then

$$\begin{aligned} & \mathbb {P} \left( \exists _{t \in [t_0+\tau v^{-2}, t_0+(\tau +T) v^{-2}]} \varvec{X}(t)> (\varvec{\nu }+\varvec{\mu }t) v \right) \\ & = v^{-\left|I \right|} \int _{\mathbb {R}^d} \mathbb {P} \left( \exists _{t \in [0, T]} \varvec{Z}_{v,\tau }(t) > (\varvec{b}- \widetilde{\varvec{b}}) \overline{\varvec{v}}v+\varvec{x} \mid \varvec{Z}_{v,\tau }(0)=\varvec{0} \right) \varphi _{\Sigma _{v,\tau }}(\widetilde{\varvec{b}}v+ \tau \varvec{\mu }v^{-1}-\varvec{x}/\overline{\varvec{v}}) d\varvec{x}. \end{aligned}$$

Define

$$\begin{aligned} \varvec{\chi }_{v,\tau }(t) := (\varvec{Z}_{v,\tau }(t) \mid \varvec{Z}_{v,\tau }(0)=\varvec{0}). \end{aligned}$$

Similarly as in the proof of Lemma 4.1, we derive that

$$\begin{aligned} \mathbb {E}\left( \varvec{\chi }_{v,\tau }(t) \right) = \left(1- r_{v,\tau }(t,0) r_{v,\tau }(0,0)^{-1}\right) \varvec{x} + \overline{\varvec{v}}\left( ( r_{v,\tau }(t,0) r_{v,\tau }(0,0)^{-1} -1) ( \widetilde{\varvec{b}}v+ \tau \varvec{\mu }v^{-1} ) -t \varvec{\mu }v^{-1}\right). \end{aligned}$$

Next, it follows that

$$\begin{aligned} r_{v,\tau }(t,0) r_{v,\tau }(0,0)^{-1} -1 = \frac{(t_0+\tau v^{-2}+tv^{-2})^{2H} - (t_0+\tau v^{-2})^{2H}}{2 (t_0+\tau v^{-2})^{2H}} - \frac{t^{2H} v^{-4H}}{2 (t_0+\tau v^{-2})^{2H}}, \end{aligned}$$

and

$$\begin{aligned} (t_0+\tau v^{-2}+tv^{-2})^{2H} - (t_0+\tau v^{-2})^{2H} = 2H (t_0+\tau v^{-2})^{2H-1} tv^{-2} + H(2H-1) (t_0+\tau ' v^{-2})^{2H-2} (tv^{-2})^2, \end{aligned}$$

with some $\tau ' \in [\tau , \tau +t]$. Some elementary calculations yield that, as $v\rightarrow \infty ,$

$$\begin{aligned} \mathbb {E}\left( \varvec{\chi }_{v,\tau }(t) \right) \rightarrow \begin{pmatrix} \left(\frac{H}{t_0}\varvec{b}_I - \varvec{\mu }_I\right) t \\ \varvec{0} _{I^c} \end{pmatrix} \end{aligned}$$

holds uniformly for $\tau$ such that $\left|\tau \right| \le T(\widehat{N}_v+1)$. Similarly to the calculations for the mean, we obtain that, as $v\rightarrow \infty ,$

$$\begin{aligned} \mathbb {E}\left( [\varvec{\chi }_{v,\tau }(t) -\mathbb {E}\left( \varvec{\chi }_{v,\tau }(t)\right) ][\varvec{\chi }_{v,\tau }(s) -\mathbb {E}\left( \varvec{\chi }_{v,\tau }(s)\right) ]^\top \right) \rightarrow \varvec{0} \end{aligned}$$

holds uniformly for $\tau$ such that $\left|\tau \right| \le T(\widehat{N}_v+1)$. Thus, as $v\rightarrow \infty ,$

$$\begin{aligned} \mathbb {P} \left( \exists _{t \in [0, T]} \varvec{Z}_{v,\tau }(t)> (\varvec{b}- \widetilde{\varvec{b}}) \overline{\varvec{v}}v +\varvec{x} \mid \varvec{Z}_{v,\tau }(0)=\varvec{0} \right) \rightarrow \mathbb {P} \left( \exists _{t \in [0, T]} \left( \frac{H}{ t_0}\varvec{b}_I-\varvec{\mu }_I\right) t >\varvec{x}_I \right) \cdot {\mathbb {I}}_{(\varvec{x}_K < \varvec{0}_K)}. \end{aligned}$$

Proceeding similarly to the proof of Lemma 4.1, we can obtain

$$\begin{aligned} & \mathbb {P} \left( \exists _{t \in [t_0+\tau v^{-2}, t_0+(\tau +T) v^{-2}]} \varvec{X}(t) > (\varvec{\nu }+\varvec{\mu }t) v \right) \\ & \sim v^{-\left|I \right|} \frac{\widetilde{\mathcal {H}}_{I}(T)}{\sqrt{ (2\pi t_0^{2H})^d \left|\Sigma \right|}} e^{-\frac{v^2}{2} g_I(t_0+\tau v^{-2})}\\ & \times \int _{\mathbb {R}^{\left|I^c \right|}} e^{-\frac{1}{2 t_0^{2H}} \varvec{x}_{I^c}^ \top (\Sigma ^{-1})_{I^cI^c} \varvec{x}_{I^c}} {\mathbb {I}}_{\left(\varvec{x}_K < - ( \tau v^{-1}) [[(\Sigma ^{-1})_{I^cI^c}]^{-1} (\Sigma ^{-1} \varvec{\mu })_{I^c}]_K \right)} d\varvec{x}_{I^c}, \end{aligned}$$

where

$$\begin{aligned} \widetilde{\mathcal {H}}_{I}(T)= & \int _{\mathbb {R}^{\left|I \right|}} e^{\frac{1}{t_0^{2H}} \varvec{w}_I^\top \varvec{x}_I} \mathbb {P} \left( \exists _{t \in [0, T]} \varvec{x}_I<-\left( \varvec{\mu }_I-\frac{H}{ t_0}\varvec{b}_I\right) t \right) d\varvec{x}_I,\\= & \frac{t_0^{2H|I|}}{\prod _{i \in I}w_i} \int _{\mathbb {R}^{\left|I \right|}} e^{ \varvec{1}_I^\top \varvec{x}_I} \mathbb {P} \left( \exists _{t \in [0, T]} \varvec{x}_I<-\frac{1}{t_0^{2H}} \textrm{diag}(\varvec{w}_I)\left( \varvec{\mu }_I-\frac{H}{ t_0}\varvec{b}_I\right) t \right) d\varvec{x}_I. \end{aligned}$$

Furthermore, since $\varvec{w}_I>\varvec{0}_I$ and

$$\begin{aligned} \varvec{1}_I^{\top } \textrm{diag}(\varvec{w}_I)\left( \varvec{\mu }_I-\frac{H}{ t_0}\varvec{b}_I\right) =\varvec{b}_I^{\top }\Sigma _{II}^{-1} \left( \varvec{b}_I'(t_0)-\frac{H}{t_0}\varvec{b}_I \right) =\frac{t_0^{2H}}{2}g_I'(t_0)=0, \end{aligned}$$

we obtain, by (Dȩbicki et al. 2020, Lemma 5.3),

$$\begin{aligned} \widetilde{\mathcal {H}}_I (T) = \frac{t_0^{2H|I|}}{\prod _{i \in I}w_i} \left( 1+\frac{T}{t_0^{2H}}\sum _{i \in I} \left( w_i \mu _i - \frac{H}{t_0} w_i b_i\right) _{-} \right), \end{aligned}$$

with $\sum _{i \in I} \left( w_i \mu _i - \frac{H}{t_0} w_i b_i\right) _{-}>0.$ This combined with Eq. (33) completes the proof. $\square$

### Proof of Lemma

4.4 The proof proceeds analogously to the proof of Lemma 4.2, however, some important changes should be applied. Hereafter the exact values of the constants $\epsilon , C_M,v_M,C_{M,\epsilon }$ and $v_{M,\epsilon }$ are not important and can be changed from line to line. Moreover, all the inequalities hold uniformly in $\tau _1,\tau _2 \in [-Mv-1,Mv]$ such that $\tau _2 -\tau _1 \ge 1$ and the constants do not depend on $\tau _1,\tau _2$.

Without loss of generality, we shall assume $I = \{1,\ldots , d\}$. Let $V \subset I$ be a non-empty set to be chosen later (*V* will not depend on $\tau _1,\tau _2$). Denote

$$\begin{aligned} & \widetilde{\varvec{X}}_v(t_1,t_2) := \begin{pmatrix} \varvec{X}_{v,V}(t_1)\\ \varvec{X}_{v,I \backslash V}(t_2) \end{pmatrix}, \ \ \varvec{X}_v(t):= \frac{\varvec{X}(t_0+tv^{-2})}{(t_0+tv^{-2})^{H}},\\ & \widetilde{R}_v(t_1,t_2,s_1,s_2) := \mathbb {E}\left( \widetilde{\varvec{X}}_v(t_1,t_2)\widetilde{\varvec{X}}^{\top }_v(s_1,s_2)\right) ,\ \ \widetilde{\Sigma }_v(t_1,t_2) := \widetilde{R}_v(t_1,t_2,t_1,t_2), \\ & r_v(t,s) := \mathbb {E}\left( \frac{B_{H,1}(t_0+tv^{-2})}{(t_0+tv^{-2})^H} \frac{B_{H,1}(t_0+sv^{-2})}{(t_0+sv^{-2})^H} \right) = \frac{(t_0+tv^{-2})^{2H}+(t_0+sv^{-2})^{2H}-|t-s|^{2H} v^{-4H}}{2 (t_0+tv^{-2})^{H}(t_0+sv^{-2})^{H}}. \end{aligned}$$

It follows that, for any $t \in [\tau _k,\tau _k +1]$ and $s \in [\tau _l,\tau _l+1]$, $k,l \in \{1,2\}$,

$$\begin{aligned} 0 \le 1-r_v(t,s)=\frac{\left((t_0+tv^{-2})^{H}-(t_0+sv^{-2})^{H}\right)^2 + (|t-s|v^{-2})^{2H} }{2 (t_0+tv^{-2})^{H}(t_0+sv^{-2})^{H}}. \end{aligned}$$

By Taylor’s formula and the fact that $H>1/2$, we have that, for any small $\epsilon >0$ there exists $v_{M,\epsilon }>0$ such that for all $v \ge v_{M,\epsilon }$ and all $t \in [\tau _k,\tau _k +1]$, $s \in [\tau _l,\tau _l+1]$, $k,l \in \{1,2\}$,

46 $$\begin{aligned} v^2\left( 1-r_v(t,s)\right)\le & \epsilon \left|t-s \right| \end{aligned}$$

47 $$\begin{aligned}\le & \epsilon +\epsilon (\tau _2-\tau _1). \end{aligned}$$

Further, denote

$$\begin{aligned} \varvec{b}_v(t_1,t_2):= \begin{pmatrix} \varvec{\zeta }_V(t_0+t_1 v^{-2}) \\ \varvec{\zeta }_{I \backslash V}(t_0+t_2 v^{-2}) \end{pmatrix} \ \ \text {with} \ \ \varvec{\zeta }(t) := \frac{\varvec{\nu }+\varvec{\mu }t}{t^H}, \end{aligned}$$

and

$$\begin{aligned} & \varvec{\chi }_v(t_1,t_2) :=v\left( \widetilde{\varvec{X}}_v(t_1,t_2) - v\varvec{b}_v(t_1,t_2)\right) +\varvec{x} \Big {|} \left( v\left( \widetilde{\varvec{X}}_v(\tau _1,\tau _2) -v \varvec{b}_v(\tau _1,\tau _2)\right) +\varvec{x}=0 \right) ,\\ & \widetilde{\varvec{w}}_v(\tau _1, \tau _2) = \widetilde{\Sigma }_v^{-1}(\tau _1,\tau _2)\varvec{b}_v(\tau _1,\tau _2). \end{aligned}$$

By the law of total probability, we obtain

48 $$\begin{aligned} \widehat{p}(\tau _1,\tau _2;v)\le & \mathbb {P} \left( \exists t_1 \in [\tau _1,\tau _1+1],t_2 \in [\tau _2,\tau _2+1]:\widetilde{\varvec{X}}_v(t_1,t_2)>v \varvec{b}_v(t_1,t_2) \right) \nonumber \\\le & v^{-|I|} \varphi _{\widetilde{\Sigma }_v(\tau _1,\tau _2)}\left( v\varvec{b}_v(\tau _1,\tau _2)\right) \int _{\mathbb {R}^{\left|I \right|}} e^{(\widetilde{\varvec{w}}_v(\tau _1, \tau _2))^{\top } \varvec{x}} \mathbb {P} \left( \exists t_1 \in [\tau _1,\tau _1+1],t_2 \in [\tau _2,\tau _2+1]:\varvec{\chi }_{v}(t_1,t_2)>\varvec{x} \right) d\varvec{x}. \end{aligned}$$

Note that, similarly to Eq. (40), $\varvec{\widetilde{w}}_v(\tau _1, \tau _2) \le \overline{ \varvec{w}}$ holds for all large *v*.

In order to find a tight upper bound for the integral in Eq. (48), similarly to the proof of Lemma 4.2, we shall verify conditions of (Ievlev 2024, Lemma 8) as stated in Eqs. (41)-(43) with *H* replaced by 1/2.

Inequality (41). Recall that $F\subseteq I=\{1,\ldots ,d\}$ is defined such that $\varvec{x}_F>\varvec{0}$ and $\varvec{x}_{I\setminus F}<\varvec{0}$ in the integral. There exist $C_M>0$, $v_M>0$ such that for all $v \ge v_M$ and all $t_1 \in [\tau _1,\tau _1+1]$ and $t_2 \in [\tau _2,\tau _2+1]$,

$$v^2 \biggl |\varvec{b}_v(t_1,t_2)-\varvec{b}_v(\tau _1,\tau _2) \biggr | \le \sup _{s \in [\tau _1,\tau _1+1] \cup [\tau _2,\tau _2+1]} \biggl |\varvec{\zeta }'_I(t_0+sv^{-2}) \biggr | \le C_M,$$

and therefore, by Eq. (47), for any small $\epsilon >0$ there exist $C_{M,\epsilon }>0$ and $v_{M,\epsilon }>0$ such that for all $v \ge v_{M,\epsilon }$ and all $t_1 \in [\tau _1,\tau _1 +1]$, $t_2 \in [\tau _2,\tau _2+1]$,

$$\begin{aligned} & \biggl |\mathbb {E}\left( \varvec{\chi }_v(t_1,t_2)\right) \biggr |= \biggl |\left( -v^2 \varvec{b}_v(t_1,t_2) + \varvec{x}\right) - \widetilde{R}_v(t_1,t_2,\tau _1,\tau _2)\widetilde{\Sigma }_v^{-1}(\tau _1,\tau _2) \left( -v^2 \varvec{b}_v(\tau _1,\tau _2)+\varvec{x}\right) \biggr | \nonumber \\ & \le v^2 \biggl | \left( \widetilde{\Sigma }_v(\tau _1,\tau _2)-\widetilde{R}_v(t_1,t_2,\tau _1,\tau _2)\right) \widetilde{\Sigma }_v^{-1}(\tau _1,\tau _2) \varvec{b}_v(\tau _1,\tau _2) \biggr |+C_{M,\epsilon }+\epsilon \left| \varvec{x}\right| \nonumber \\ & \le C_{M,\epsilon }+\epsilon ((\tau _2-\tau _1)+\left| \varvec{x}\right| ), \end{aligned}$$

where in the last inequality we use (here $||A|| = \max _{i,j} \left|a_{ij} \right|$ for a matrix *A*, and Eq. (47) is applied)

$$\begin{aligned} {v^2\left.{\&\#XArrowvert;}\widetilde{\Sigma }_v(\tau _1,\tau _2)-\widetilde{R}_v(t_1,t_2,\tau _1,\tau _2) \right.{\&\#XArrowvert;}}= & \left.{\&\#XArrowvert;}\begin{array}{cc} v^2(r_v(\tau _1,\tau _1)- r_v(t_1,\tau _1))\Sigma _{V,V} & v^2(r_v(\tau _1,\tau _2)- r_v(t_1,\tau _2)) \Sigma _{V,I \backslash V}\\ v^2(r_v(\tau _2,\tau _1)-r_v(t_2,\tau _1))\Sigma _{I \backslash V,V} & v^2(r_v(\tau _2,\tau _2)-r_v(t_2,\tau _2))\Sigma _{I \backslash V, I \backslash V} \end{array}\right.{\&\#XArrowvert;}\\\le & C_{M,\epsilon }+\epsilon (\tau _2-\tau _1). \end{aligned}$$

Thus, we conclude that for any small $\epsilon >0$ there exist $C_{M,\epsilon }>0$ and $v_{M,\epsilon }>0$ such that for $v \ge v_{M,\epsilon }$, inequality Eq. (41) with $H=1/2$ holds.

Inequality Eq. (42). For any $t_1 \in [\tau _1,\tau _1 +1]$ and $t_2 \in [\tau _2,\tau _2+1]$, we have, for the covariance matrix of $\varvec{\chi }_{v}(t_1,t_2)$, that

$$\begin{aligned} \text {Cov} (\varvec{\chi }_{v}(t_1,t_2)) )=v^2 \left( \widetilde{R}_v(t_1,t_2,t_1,t_2) - \widetilde{R}_v(t_1,t_2,\tau _1,\tau _2)\widetilde{\Sigma }_v^{-1}(\tau _1,\tau _2) \widetilde{R}_v(\tau _1,\tau _2,t_1,t_2) \right). \end{aligned}$$

Using Eq. (47) and some calculations, we can show that, for any small $\epsilon >0$ there exist $C_{M,\epsilon }>0$ and $v_{M,\epsilon }>0$ such that for all $v \ge v_{M,\epsilon }$ and all $t_1 \in [\tau _1,\tau _1 +1]$, $t_2 \in [\tau _2,\tau _2+1]$,

$$\begin{aligned} || \text {Cov} (\varvec{\chi }_{v}(t_1,t_2)) ) || \le C_{M,\epsilon }+\epsilon (\tau _2-\tau _1). \end{aligned}$$

Thus, for any small $\epsilon >0$ there exist $C_{M,\epsilon }>0$ and $v_{M,\epsilon }>0$ such that for $v \ge v_{M,\epsilon }$ inequality Eq. (42) with $H=1/2$ holds.

Inequality Eq. (43). We have, using Eq. (31), that, for any $F\subseteq I,$

49 $$\begin{aligned} \text {Var}\left( \overline{\varvec{w}}_F^{\top } \chi _{v,F}(t_1,t_2)-\overline{\varvec{w}}_F^{\top } \chi _{v,F}(s_1,s_2)\right) \le v^2 \text {Var}\left( \overline{\varvec{w}}_F^{\top } \widetilde{\varvec{X}}_{v,F}(t_1,t_2)-\overline{\varvec{w}}_F^{\top } \widetilde{\varvec{X}}_{v,F}(s_1,s_2)\right) . \end{aligned}$$

Further, it follows that there exist $C_M>0$, $v_M>0$ such that for all $v \ge v_M$ and all $t_1,s_1 \in [\tau _1,\tau _1+1]$, $t_2,s_2 \in [\tau _2,\tau _2 + 1]$,

50 $$\begin{aligned} & v^2 \text {Var}\left( \overline{\varvec{w}}_F^{\top } \widetilde{\varvec{X}}_{v,F}(t_1,t_2)-\overline{\varvec{w}}_F^{\top } \widetilde{\varvec{X}}_{v,F}(s_1,s_2)\right) \nonumber \\ & \le 2v^2 \textrm{Var} \left( \overline{\varvec{w}}_{F\cap V}^{\top } {\varvec{X}}_{v, F \cap V}(t_1) - \overline{\varvec{w}}_{F\cap V}^{\top } {\varvec{X}}_{v, F \cap V}(s_1)\right) \nonumber \\ & \quad + 2v^2 \textrm{Var} \left( \overline{\varvec{w}}_{F \cap (I \backslash V)}^{\top } {\varvec{X}}_{v, F \cap (I \backslash V)}(t_2) - \overline{\varvec{w}}_{F \cap (I \backslash V)}^{\top } {\varvec{X}}_{v, F \cap (I \backslash V)}(s_2)\right) \nonumber \\ & \le C_M(|t_1-s_1|+|t_2-s_2|), \end{aligned}$$

where the last inequality follows by an application of Eq. (46). The above confirms that inequality Eq. (43) with $H=1/2$ is satisfied.

To sum up, we have checked that the conditions of (Ievlev 2024, Lemma 8) are satisfied, and therefore, for any small $\epsilon >0$ there exist $C_{M,\epsilon },v_{M,\epsilon }>0$ such that for all $v \ge v_{M,\epsilon }$,

51 $$\begin{aligned} \int _{\mathbb {R}^{\left|I \right|}} e^{(\widetilde{\varvec{w}}_v(\tau _1,\tau _2))^{\top } \varvec{x}} \mathbb {P} \left( \exists t_1 \in [\tau _1,\tau _1+1],t_2 \in [\tau _2,\tau _2+1]:\varvec{\chi }_{v}(t_1,t_2)>\varvec{x} \right) d\varvec{x} \le e^{C_{M,\epsilon }+\epsilon (\tau _2-\tau _1)}. \end{aligned}$$

It remains to estimate $\varphi _{\widetilde{\Sigma }_v(\tau _1,\tau _2)}\left( v \varvec{b}_v(\tau _1,\tau _2)\right)$. By Eq. (47), we have, for any small $\epsilon >0$ there exist $C_{M,\epsilon },v_{M,\epsilon }>0$ such that, for all $v \ge v_{M,\epsilon }$,

52 $$\begin{aligned} \begin{aligned} \varphi _{\widetilde{\Sigma }_v(\tau _1,\tau _2)}\left( v \varvec{b}_v(\tau _1,\tau _2)\right)&\le C_{M,\epsilon }\exp \left( -\frac{1}{2} v^2 \varvec{b}_v^{\top }(\tau _1,\tau _2) \widetilde{\Sigma }_v^{-1}(\tau _1,\tau _2) \varvec{b}_v(\tau _1,\tau _2) \right) \\&\le C_{M,\epsilon } \exp \left( -\frac{1}{2} v^2 \varvec{b}_v^{\top }(\tau _1,\tau _2) \Sigma ^{-1}\varvec{b}_v(\tau _1,\tau _2) +\epsilon (\tau _2-\tau _1) \right) . \end{aligned} \end{aligned}$$

On the other hand, we have by Lagrange’s mean value theorem that, for some $x \in [\tau _1,\tau _2]$,

$$\begin{aligned} \varvec{b}_v^{\top }(\tau _1,\tau _2)\Sigma ^{-1} \varvec{b}_v(\tau _1,\tau _2)= \varvec{b}_v^{\top }(\tau _1,\tau _1)\Sigma ^{-1} \varvec{b}_v(\tau _1,\tau _1) +2v^{-2}(\tau _2-\tau _1) \varvec{b}_v^{\top }(\tau _1,x)\Sigma ^{-1} \begin{pmatrix} \varvec{0}_V\\ \varvec{\zeta }'_{I \backslash V}(t_0+xv^{-2}) \end{pmatrix}. \end{aligned}$$

Therefore, for any small $\epsilon >0$, there exists $v_{M,\epsilon }$ such that, for all $v \ge v_{M,\epsilon }$

53 $$\begin{aligned} v^2 \left| \varvec{b}_v^{\top }(\tau _1,\tau _2)\Sigma ^{-1} \varvec{b}_v(\tau _1,\tau _2)- g_I(t_0+\tau _1 v^{-2}) -2v^{-2}(\tau _2-\tau _1)\varvec{\zeta }_I(t_0)^{\top }\Sigma _{II}^{-1} \begin{pmatrix} \varvec{0}_V\\ \varvec{\zeta }'_{I \backslash V}(t_0) \end{pmatrix} \right| \le \epsilon (\tau _2-\tau _1). \end{aligned}$$

Now take $V = \{i \in I: \zeta '_i(t_0) \le 0\}$. Since $\varvec{\zeta }'_I(t_0) \ne \varvec{0}_I$ (due to $H\varvec{\nu }_I \ne (1-H)t_0\varvec{\mu }_I$), $\Sigma _{II}^{-1} \varvec{\zeta }_I(t_0)> \varvec{0}_I$ and $2\varvec{\zeta }_I(t_0)^{\top }\Sigma _{II}^{-1}\varvec{\zeta }'_I(t_0)=g'_I(t_0)=0$, we know that both *V* and $I \backslash V$ are non-empty. Hence, using once again that $\Sigma _{II}^{-1} \varvec{\zeta }_I(t_0)> \varvec{0}_I$, we obtain

54 $$\begin{aligned} \varvec{\zeta }_I(t_0)^{\top } \Sigma _{II}^{-1} \begin{pmatrix} \varvec{0}_V\\ \varvec{\zeta }'_{I \backslash V}(t_0) \end{pmatrix} >0. \end{aligned}$$

Therefore, combining Eqs. (52)-(54) we have that, for any small $\epsilon >0$ there exist $C, C_{M,\epsilon },v_{M,\epsilon }>0$ such that for all $v \ge v_{M,\epsilon }$,

$$\begin{aligned} \varphi _{\widetilde{\Sigma }_v(\tau _1,\tau _2)}(v\varvec{b}_v(\tau _1,\tau _2))\le & C_{M,\epsilon } \exp \left( -\frac{v^2}{2}g_I(t_0+\tau _1 v^{-2})- C(\tau _2-\tau _1)\right) \\\le & C_{M,\epsilon } \exp \left( -\frac{v^2}{2}g_I(t_0)- C(\tau _2-\tau _1)\right) . \end{aligned}$$

This, combined with Eqs. (48) and (51), establishes the claim of the lemma. $\square$
